# Effect of a Community-Based Gender Norms Program on Sexual Violence Perpetration by Adolescent Boys and Young Men: A Cluster Randomized Clinical Trial

**DOI:** 10.1001/jamanetworkopen.2020.28499

**Published:** 2020-12-22

**Authors:** Elizabeth Miller, Kelley A. Jones, Alison J. Culyba, Taylor Paglisotti, Namita Dwarakanath, Michael Massof, Zoe Feinstein, Katie A. Ports, Dorothy Espelage, Julie Pulerwitz, Aapta Garg, Jane Kato-Wallace, Kaleab Z. Abebe

**Affiliations:** 1 Division of Adolescent and Young Adult Medicine, Department of Pediatrics, UPMC Children’s Hospital of Pittsburgh, University of Pittsburgh School of Medicine, Pittsburgh, Pennsylvania; 2 Division of Violence Prevention, National Center for Injury Prevention and Control, Centers for Disease Control and Prevention, Atlanta, Georgia; 3 University of North Carolina at Chapel Hill School of Education, Chapel Hill; 4 Population Council, Washington, District of Columbia; 5 Promundo-US, Washington, District of Columbia; 6 Division of General Internal Medicine, University of Pittsburgh School of Medicine, Pittsburgh, Pennsylvania

## Abstract

**Importance:**

Engaging adolescent boys and young men in preventing violence against women is a potentially impactful public health strategy.

**Objective:**

To evaluate the effectiveness of a community-based, gender-transformative program (ie, Manhood 2.0) on perpetration of gender-based violence by adolescent boys and young men.

**Design, Setting, and Participants:**

In this unblinded cluster randomized clinical trial, neighborhoods were designated as the unit of clustering (1:1 allocation). Three-month (ie, time point 2 [T2]) and 9-month (ie, time point 3 [T3]) follow-ups were conducted. The trial took place in 20 Pittsburgh, Pennsylvania, neighborhoods and 1 centrally located site with concentrated disadvantage. Pittsburgh-based adolescent boys and young men (ages 13 to 19 years) were recruited between July 27, 2015, and June 5, 2017, through youth-serving organizations and community-based alternatives to residential placement for juvenile justice–involved youth. Intention-to-treat analysis was conducted from June 2018 to November 2019.

**Interventions:**

Manhood 2.0, an international program adapted for adolescent boys and young men in US urban communities, encourages these individuals to challenge gender norms that foster violence against women and unhealthy sexual relationships. Individuals in the control population received job-readiness training. Each program was 18 hours.

**Main Outcomes and Measures:**

The primary outcome was change in participant-level perpetration of sexual violence (SV) or adolescent relationship abuse (ARA) at T3.

**Results:**

Among 866 participants, 465 individuals (54%) enrolled in 11 intervention clusters and 401 individuals (46%) enrolled in 10 control clusters. In the intervention group, 325 participants (70%) were analyzed at T2 and 334 participants (72%) were analyzed at T3; in the control group, 262 participants (65%) were analyzed at T2 and 301 participants (75%) were analyzed at T3. Mean (SD) age was 15.5 (1.6) years; 609 participants (70%) self-identified as non-Hispanic Black, and 178 (20%) self-identified as Hispanic, multiracial, or other race/ethnicity other than White. Among individuals in the intervention group, 296 participants (64%) reported any SV or ARA perpetration at baseline, and 173 participants (52%) reported any SV or ARA perpetration at T3. Among individuals in the control group, 213 participants (53%) reported any SV or ARA perpetration at baseline, and 124 participants (41%) reported any SV or ARA perpetration at T3). The difference in reduction between groups was not significant. There was no evidence of an intervention effect for the primary outcome (adjusted odds ratio [OR], 1.32; 95% CI, 0.86-2.01; *P* = .20).

**Conclusions and Relevance:**

The findings from this evaluation of a community-based gender-transformative program for adolescent boys and young men did not show a significant intervention effect in reducing SV or ARA perpetration between Manhood 2.0 and a job-readiness control program. Combining gender-transformative approaches with job-readiness programs may be relevant for violence prevention in low-resource urban settings. Attention to improving implementation and strategies to sustain such community-based efforts are needed.

**Trial Registration:**

ClinicalTrials.gov Identifier: NCT02427061

## Key Points

**Question:**

Does a gender-transformative program, compared with job-readiness training, have a greater effect on rates of perpetration of gender-based violence by adolescent boys and young men living in US urban neighborhoods with concentrated disadvantage?

**Findings:**

In this cluster randomized clinical trial of 866 adolescent boys and young men, the proportion of individuals reporting perpetrating gender-based violence in the past 9 months was reduced among individuals receiving a gender-transformative program and those receiving job-readiness training; the difference in reduction between the 2 groups was not significant.

**Meaning:**

These findings suggest that testing of efforts that combine critical reflections about masculinities with job-readiness training for adolescent boys and young men in lower-resource neighborhoods is needed.

## Teaser

This cluster randomized clinical trial compares the effects of a gender norms program with those of a job-readiness program on sexual violence perpetration by adolescent boys and young men in disadvantaged neighborhoods.

## Introduction

At least 1 in 3 women and nearly 1 in 5 men in the United States experiences sexual violence (SV) in their lifetime.^1^ Among adolescents, experiences of nonpartner SV often overlap with experience of adolescent relationship abuse (ARA; ie, physical, sexual, or emotional abuse by a partner).^2^ Exposure to such violence is associated with poor health, including suicidality, depression, substance use, unintended pregnancy, and sexually transmitted infections.^3,4^

Prevention of SV and ARA requires modifying the behaviors of perpetrators.^5^ One modifiable factor may be gender-inequitable attitudes among adolescent boys and young men, as these attitudes are associated with violence experienced by women.^6^ Social contexts in which boys and men demonstrate negative attitudes toward girls and women, endorse prejudices regarding sexuality, and condone abuse are associated with an environment in which the perpetration of SV and ARA is prevalent.^7^ Adolescent sexual health programs that incorporate discussions of gender norms and power are associated with positive shifts in gender attitudes, increased condom use, and decreased male-perpetrated partner violence.^8,9^ Additionally, bystander-based interventions encouraging youth to intervene when witnessing peers’ harmful behaviors are associated with reduced SV and ARA.^5^ Thus, such gender-transformative approaches, which encourage development of healthy masculinities (ie, respectful sexual behaviors and exploration of masculinity norms that promote gender equity), combined with bystander skills development may be helpful in improving sexual health and reducing SV and ARA perpetration.

To our knowledge, this cluster-randomized trial is the first study in the United States to test effectiveness among adolescent boys and young men of a community-based gender-transformative program (ie, one that challenges homophobia and gender-based harassment, questions harmful masculinity norms, and demonstrates more respectful behaviors) combined with bystander and healthy sexuality skills to prevent SV and ARA. The cluster design was performed at the neighborhood level to mitigate contamination. This study compared the effectiveness of Manhood 2.0 with that of job-readiness training in reducing participants’ self-reported SV and ARA perpetration (ie, the primary outcome)*.* We hypothesized that Manhood 2.0 would result in a greater decrease in any SV or ARA perpetration compared with job-readiness training. Secondary outcomes included participants’ gender-equitable attitudes, recognition of abusive behaviors, intentions to intervene with peers, condom self-efficacy and contraceptive use attitudes, and positive and negative bystander behavior.

## Methods

This cluster study protocol and all changes were approved by the University of Pittsburgh’s institutional review board. The University of Pittsburgh Human Subjects Research Protection Office approved a waiver of parental permission because the study was considered minimal risk and because requiring parental consent would have been an additional barrier for youth whose adult caregivers may not have been available to provide consent. Research assistants reviewed verbal assent and consent and answered participants’ questions at each time point (Trial Protocol in Supplement 1). This trial adhered to the Consolidated Standards of Reporting Trials (CONSORT) reporting guideline.

### Intervention Descriptions

Manhood 2.0 was the first US adaptation of Program H, a gender-transformative curriculum for adolescent boys and young men in Brazil.^10,11^ Modifications to produce Manhood 2.0, described in detail elsewhere,^10,11^ included addition of content on racial justice, social media, and pornography.

Each group involved an 18-hour curriculum divided into 6 sessions of 3 hours each delivered once or twice a week; these were delivered among 45 unique intervention groups and 41 unique control groups, generally consisting of 8 to 12 individuals, between July 2015 and August 2017. Facilitators were community leaders from these neighborhoods, providing significant dosage via content repetition and skills practice.^12^

#### Intervention and Control

The intervention, Manhood 2.0, prompted participants to discuss healthy relationships and sexuality, identify gender norms, recognize disrespectful behaviors, and practice positive bystander interventions when witnessing negative peer behaviors, as described elsewhere.^11^ The control, Jump Start Success: Work Readiness and Career Exploration Training,^13^ is a widely used job-readiness curriculum. Investigators chose this intervention to ensure comparable recruitment numbers between treatment groups. While job readiness is considered promising for youth violence prevention,^14^ this program was not expected to have robust effects on SV outcomes, as aspects of SV are distinct from youth violence.^5,15^

#### Setting

The study population was high school–age boys and young men from racially segregated neighborhoods in Pittsburgh, Pennsylvania. These neighborhoods had high prevalence of poverty, school suspension, and community violence, as described elsewhere.^11^

### Randomization

We identified 20 neighborhoods and 1 centrally located site as clusters, and the principal investigator (E.M.) enrolled these 21 clusters (Figure). Clusters were randomly allocated (1:1 ratio) to intervention or control groups by the study statistician (K.Z.A.) using permuted blocks in Stata/SE statistical software version 14 (StataCorp), with allocations stratified by lead agency (ie, Young Men’s Christian Association, Urban League, or other). Participants were recruited to the study after randomization, so participants and research staff were aware of a site’s allocation (ie, Manhood 2.0 or job-readiness site) before enrolling.

**Figure.**
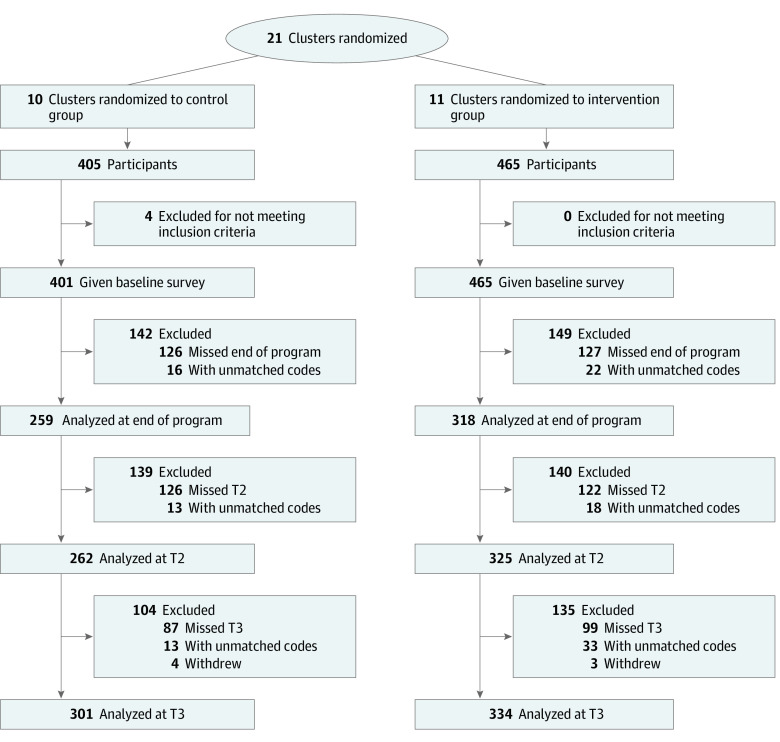
Study Participant Recruitment and Retention Flowchart T2 indicates time 2 (approximately 3 months after end of program); T3, time 3 (approximately 9 months after end of program); and unmatched codes, unable to match participant-generated secret code.

### Recruitment and Retention

Eligible participants were ages 13 to 19 years, had self-identified as male, and had stated a willingness to participate in their neighborhood’s program. Participants were recruited from July 27, 2015, to June 5, 2017. Recruitment relied on community members and respondent-driven sampling.^11^ Because of these recruitment methods, we were unable to assess the number of boys and young men approached to participate.

Participants completed surveys before the first session (ie, baseline), at the end of the program, and approximately 3 months (ie, time point 2 [T2]) and 9 months (ie, time point 3 [T3]) after the end of the program; follow-up was collected August 12, 2015, through May 18, 2018. All baseline surveys were completed in person using tablets at community sites. End of program, T2, and T3 surveys were completed in community locations or remotely (eFigure in Supplement 2). Participants were offered up to $100 in incentives. Each survey was anonymously linked across time points by a previously tested youth-generated code, designed to protect participant identity and increase respondent honesty.^16^

Protocol changes made 2 months after study commencement included expanding age range, from 14 to 19 years to 13 to 19 years, and adjusting incentives so participants received compensation after each session rather than only with surveys. At 4 months after commencement, staff began offering remote follow-up surveys to participants not available in person. Finally, 11 months after trial commencement, respondent-driven sampling was initiated to promote recruitment.

### Measures

Participant age, race/ethnicity, school grade, school status, country of birth, and juvenile justice involvement and highest education attained by participant’s parent were self-reported. Race/ethnicity was assessed owing to high racial segregation in Pittsburgh’s low-resource neighborhoods.

At baseline and T3, participants reported whether they had perpetrated any SV or ARA in the previous 9 months. The primary outcome measure was any SV or ARA perpetration, recorded as a yes response to questions about acts in 7 subdomains (Table 1).^2,17,18,19,20,21^ As an exploratory outcome, all yes responses to individual items were summed to create an SV and ARA summary score (range, 0-27). Each subdomain in Table 1 was also assessed as an exploratory outcome: physical or sexual intimate partner violence, nonpartner SV, any SV, dating abuse, sexual harassment, cyber–sexual abuse, and incapacitated sex.

**Table 1.** Violence Perpetration Measures Used to Evaluate Manhood 2.0

Domain(s)	Items	Response options
Physical/sexual intimate partner violence^17^		
Any sexual violence	“Have YOU done any of the following to someone you were in a relationship with (like he or she was your partner/girlfriend/boyfriend, you were dating or going out with them) or hooking up with: …hit, pushed, slapped, choked, or otherwise physically hurt someone you were going out with or hooking up with? (include such things as hitting, slamming into something, or injuring with an object or weapon)? [If yes] In the past nine months, have you hit, pushed, slapped, choked or otherwise physically hurt someone you were going out with or hooking up with? (include such things as hitting, slamming into something, or injuring with an object or weapon)? …used physical force or threats to make someone you were going out with or hooking up with have sex (vaginal, oral, or anal sex) when they didn't want to? [If yes] In the past 9 months (since [insert month]), have you used physical force or threats to make someone you were going out with or hooking up with have sex (vaginal, oral, or anal sex) when they didn't want to? …had sex with someone you were going out with or hooking up with when they didn't want to or because you made them feel like they didn't have a choice (even though you did not use physical force or threats)? [If yes] In the past 9 months (since [insert month]), have you had sex with someone you were going out with or hooking up with when they didn't want to, or made them feel like they didn't have a choice, even though you did not use physical force or threats?”	“Yes” or “no” to each Modeled as yes to any lifetime (baseline only) and yes to any past 9 months
Dating abuse^18^	“Have YOU done any of the following to someone you were going out with (like he or she was your partner/girlfriend/boyfriend, you were dating them) or hooking up with: Spread rumors about their sexual reputation, like telling people they're ‘easy’? Convinced them to have sex, after they had said no a few times? Made them have sex when they didn’t want to? Physically hurt them (like shoving, grabbing, slapping, punching, choking)? Threatened to hurt them if they didn't do what you wanted them to do? Yelled at them or destroyed something that belonged to them? Called them names, like ugly or stupid? Told them not to talk to others or told them who they could hang out with? Showed friends or posted pictures of them naked or doing something sexual? Talked about what you and your partner do sexually with your friends or peers?”	Baseline: “No, I have never done this to someone I was in a relationship with” T2: “Yes, I have done this in the past 9 months” or “Yes, I have done this, but not in the past 9 months” T3: “Yes” or “no” Modeled as summary score for lifetime (baseline only) and past 9 months
Nonpartner sexual violence and any sexual violence^17^	“Now think about experiences you may have had with people who you were NOT going out with or hooking up with (this could include strangers, friends, family, or people you don't know well). Please tell us whether YOU have ever done these things to anyone you were NOT going out or hooking up with: …used physical force or threats to make someone you were not going out with or hooking up with have sex (vaginal, oral, or anal sex) with you when they didn't want to? [If yes] In the past 9 months (since [insert month]), have you ever used physical force or threats to make someone you were NOT going out or hooking up with have sex (vaginal, oral, or anal sex) with you when they didn't want to? …insisted that someone you were not going out with or hooking up with have sex (vaginal, oral, or anal sex) when they didn't want to, without using force or threats? [if yes] In the past 9 months (since [insert month]), have you ever insisted that someone you were NOT going out with or hooking up with have sex (vaginal, oral, or anal sex) when they didn't want to, without using force or threats?”	“Yes” or “no” to each Modeled as yes to any lifetime (baseline only) and yes to any past 9 months
Sexual harassment^19^	“In the past 9 months since [insert month], how often have you done the following things to someone when they did not want you to? Made unwelcome sexual comments, jokes, gestures, or looks? Showed, gave, or left sexual pictures, drawings, messages, or notes? Spread sexual rumors about them? Touched, grabbed, or pinched them in a sexual way? Forced them to kiss you?”	4-Point scale for frequency: 1, “never”; 2, “a few times”; 3, “once or twice a week”; and 4, “every day or almost every day” Modeled as a summary score (never = 0; any other response = +1)
Cyber–sexual abuse^2^	“In the past 9 months since [insert month], how often did you do the following to someone? How often did you… …try to get them to talk about sex when they did not want to? …ask them to do something sexual that they did not want to do? …post or publicly share a nude or semi-nude picture of them… …using mobile apps, social networks, texts, or other digital communication?”	4-Point scale for frequency: 1, “never”; 2, “a few times”; 3, “once or twice a week”; and 4, “every day or almost every day” Modeled as yes for any response of “a few times” or more often
Incapacitated sex,^20,21^ ie, partner intoxicated		
	“In the past 9 months since [insert month], have you done something sexual with someone when they were too drunk or high to stop you (this can include kissing, touching, fingering them, or having intercourse)? In the past 9 months since [insert month], have you given someone alcohol or drugs on purpose so you could do something sexual with them (this can include kissing, touching, fingering them, or having intercourse)?”	“Yes” or “no” Modeled as yes to any for incapacitated sex

Secondary outcomes included gender-equitable attitudes, recognition of ARA, intention to intervene with peers, condom negotiation self-efficacy, and attitudes related to condom and contraceptive use. A 13-item scale modified from previous studies^18,22,23^ measured participants’ views on gender norms (Cronbach α = .64). A 12-item scale^24^ measured the ability of participants to recognize harmful actions committed against a partner as abusive (Cronbach α = .94). An 8-item scale measured the likelihood that a participant would intervene when witnessing harmful behaviors among male peers (Cronbach α = .94). A 5-item scale^25^ was used to assess participants’ confidence in negotiating condom use with a partner (Cronbach α = .50). A 10-item scale^26,27,28,29^ with positive and negative statements was used to assess participants’ views on use of condoms and contraceptives (Cronbach α = .47). For secondary outcomes, mean scores were calculated for all scales unless otherwise specified, with higher scores indicating the desired outcome. All secondary outcomes are described in greater detail elsewhere.^11^

In further analysis of bystander intervention behaviors, participants were asked, via a self-administered questionnaire, whether they had witnessed any of 9 disrespectful or abusive behaviors committed by peers in the previous 3 months and what their response was (responses included positive options, such as “told the person in private that acting like that was not okay,” and negative options, such as “laughed or went along with it”).^18^ For positive interventions, all behaviors with at least 1 positive response were summed, creating a maximum summary score of 9, with not witnessing and no response coded as zero (range, 0-9). A similar calculation was performed for negative responses.

### Statistical Analysis

Power and sample size are discussed in detail elsewhere.^11^ We calculated the detectable difference in our primary outcome using methods that assumed a fixed number of clusters and 41 individuals per cluster.^30^ Based on conservative estimates from our previous work,^18,31^ we assumed a within-cluster intraclass correlation coefficient (ICC) of 0.01, a baseline SV perpetration rate of 20% in the control group, and a retention rate at T3 of 80%. With 866 participants across 21 clusters, we had adequate power (80%) to detect a 42% relative decrease (absolute decrease, 8.3 percentage points) in SV perpetration due to the intervention. For secondary outcomes, we observed within-cluster ICCs ranging from 0.006 to 0.01 in previous studies.^2,17,18,31^ Thus, we were adequately powered (≥80%) to detect standardized mean differences between study groups of as small as 0.23.

To assess missing data, we tested differences in attrition with Wald log-linear χ^2^ tests and linear regressions comparing baseline demographic characteristics and secondary outcome values between individuals who completed the primary outcome at T3 and those who did not, accounting for neighborhood-level clustering.^11^ We used generalized linear mixed models to obtain intention-to-treat estimates of intervention effects in the primary outcome at T3 compared with control groups. Models included variables for baseline perpetration, treatment group, randomization stratification variable (ie, neighborhood site type: Young Men’s Christian Association, Urban League, or other), and random effects for within-person and within-neighborhood clustering. We specified a priori that demographic characteristics differing by treatment group would be included as covariates, so race/ethnicity was added into the models. Secondary outcomes, including variables for the outcome at baseline and all other covariates mentioned, were similarly modeled. Models with convergence errors used a collapsed race/ethnicity variable (ie, Black, Hispanic, or other). All analyses were intention-to-treat with data available for all clusters.

We conducted an exploratory, prespecified, intensity-adjusted analysis that reflected actual delivery of the intervention. A continuous intensity score was calculated for each program delivery to a group of participants (86 rounds total). Scores consisted of fidelity data recorded by research assistants observing each session and round-level attendance percentages. The fidelity data were based on proportion of completed activities and extent to which objectives were met (ie, poorly to exceptionally on a 5-point Likert scale) for core curriculum activities. Attendance scores were calculated as percentage of total sessions attended by participants, calculated as means at level of round. This continuous score of intensity replaced the binary intervention variable in generalized linear mixed models. Control participants were coded as zero for the intensity score.

An ad hoc exploratory analysis tested within-arm differences in outcomes using Wald log-linear χ^2^ tests (for categorical variables) and univariate linear regressions (for continuous variables), all accounting for clustered data. Based on our a priori statistical analysis plan,^11^ the primary hypothesis was tested at the 5% type I error level and no adjustments were made for multiple testing with secondary outcomes; *P* values were 2-sided. SAS statistical software version 9.4 (SAS Institute) was used for all statistical analyses. Intention-to-treat analysis was conducted from June 2018 to November 2019.

## Results

Among 866 participants in 11 intervention neighborhoods, 465 participants (54%) enrolled, of whom 325 participants were analyzed at T2 (70% retention) and 334 participants were analyzed at T3 (72% retention). In 10 control neighborhoods, 401 participants (46%) enrolled, among whom 262 participants were analyzed at T2 (65% retention) and 301 participants were analyzed at T3 (75% retention) (Figure). Among 866 participants, 609 individuals (70%) identified as Black and 178 individuals (21%) identified as Hispanic, multiracial, or other race/ethnicity other than White (Table 2); 758 individuals (88%) were born in the United States, and mean (SD) age of participants was 15.5 (1.6) years, with 163 participants (19%) in middle school and 533 participants (62%) in high school.

**Table 2.** Baseline Participant Demographic Characteristics

Characteristic	No. (%)^a^		
	Total (N = 866)	Intervention group (n = 465)	Control group (n = 401)
Age, mean (SD), y	15.5 (1.6)	15.6 (1.5)	15.3 (1.7)
Race/ethnicity^b^			
Hispanic	53 (6)	16 (4)	37 (9)
Black	609 (70)	316 (68)	293 (73)
White	29 (3)	22 (5)	7 (2)
Multiracial	55 (6)	37 (8)	18 (5)
Other	70 (8)	48 (10)	22 (5)
Born in the United States			
Yes	758 (88)	409 (88)	349 (87)
No	49 (6)	31 (7)	18 (5)
Education status			
Currently in school	734 (85)	398 (86)	336 (84)
Not in school			
Completed high school degree	28 (3)	14 (3)	14 (4)
Did not complete high school degree	42 (5)	19 (4)	23 (6)
Current grade level^c^			
8th	163 (22)	75 (19)	88 (26)
9th	180 (25)	100 (25)	80 (24)
10th	151 (21)	88 (22)	63 (19)
11th	130 (18)	75 (19)	55 (16)
12th	72 (10)	44 (11)	28 (8)
Finished high school or received GED	9 (1)	5 (1)	4 (1)
College	6 (1)	5 (1)	4 (1)
Juvenile justice involvement			
Yes	103 (12)	53 (11)	50 (12)
No	763 (88)	412 (89)	351 (88)
Parents’ or guardians’ highest education			
Did not complete high school	378 (44)	194 (42)	184 (46)
Completed high school or GED	149 (17)	79 (17)	70 (17)
Some college	66 (8)	35 (8)	31 (8)
College degree or higher	208 (24)	120 (26)	88 (22)

Abbreviation: GED, General Educational Development.

^a^ Percentages may not sum to 100 because of small amounts of missing data.

^b^ All race/ethnicity categories except for Hispanic are non-Hispanic (eg, non-Hispanic Black).

^c^ Among those currently in school.

Participants who completed the primary outcome at T3 had higher mean (SD) gender-equitable attitudes at baseline compared with participants who did not (3.41 [0.51] vs 3.31 [0.49]). Additionally, 103 individuals (12%) were involved in the juvenile justice system, and these participants were less likely to complete T3 compared with noninvolved participants (54 participants [52%] vs 569 participants [75%]). No other differences were found comparing individuals who completed the study with those who did not (eTable in Supplement 2).

Table 3 shows outcome values at each time point by treatment group; Table 4 shows intervention effects comparing intervention to control. There was no evidence of an intervention effect for the primary outcome (adjusted odds ratio [OR], 1.32; 95% CI, 0.86-2.01; *P* = .20). Among individuals in the intervention group, 296 participants (64%) reported any SV or ARA perpetration at baseline and 173 participants (52%) reported any SV or ARA perpetration at T3. Among individuals in the control group, 213 participants (53%) reported any SV or ARA perpetration at baseline and 124 participants (41%) reported any SV or ARA perpetration at T3. Participants in intervention groups, compared with participants in control groups, had higher reported mean summary scores for SV or ARA perpetration (risk ratio, 1.47; 95% CI, 1.05-2.04; *P* = .02) and were more likely to report any cyber–sexual abuse (OR, 1.71; 95% CI, 1.04-2.82; P = .04). No other differences in SV or ARA perpetration by group were found.

**Table 3.** Within-Group Differences in Outcome Values at Baseline and Follow-up

Outcome	Baseline		T2				T3			
	Intervention (n = 465)	Control (n = 401)	Intervention (n = 325)	*P* value^a^	Control (n = 262)	*P* value^a^	Intervention (n = 334)	*P* value^a^	Control (n = 301)	*P* value^a^
**Primary outcome**										
Any SV or ARA, No. (%)^b^	296 (64)	213 (53)	NA	NA	NA	NA	173 (52)	.02	124 (41)	<.001
**Additional measures, No. (%)**										
SV or ARA summary score (27 items)										
Mean (SE)	2.77 (0.13)	2.53 (0.14)	NA	NA	NA	NA	2.41 (0.35)	.25	1.68 (0.10)	<.001
No. (IQR)	1 (0-4)	1 (0-4)	NA	NA	NA	NA	1 (0-4)	NA	0 (0-2)	NA
Any SV (partner or nonpartner)	33 (7)	30 (7)	NA	NA	NA	NA	44 (13)	.009	30 (10)	.19
Any ARA^c^	104 (31)	86 (31)	NA	NA	NA	NA	40 (16)	.001	30 (14)	.03
Any physical or sexual intimate partner violence^c^	21 (6)	21 (8)	NA	NA	NA	NA	9 (4)	.03	7 (3)	.06
Any nonpartner SV	24 (5)	19 (5)	NA	NA	NA	NA	40 (12)	.003	25 (8)	.11
Any sexual harassment	239 (51)	176 (44)	NA	NA	NA	NA	137 (41)	.12	101 (34)	.001
Incapacitated sex										
Any	54 (12)	43 (11)	NA	NA	NA	NA	38 (11)	.89	21 (7)	.05
Partner intoxicated	43 (9)	28 (7)	NA	NA	NA	NA	26 (8)	.54	15 (5)	.20
Provided partner substance	20 (4)	27 (7)	NA	NA	NA	NA	18 (5)	.37	8 (3)	.01
Any cyber–sexual abuse	118 (25)	99 (25)	NA	NA	NA	NA	87 (26)	.80	49 (16)	.02
**Secondary outcomes, mean (SE)**										
Gender-equitable attitudes	3.38 (0.04)	3.40 (0.04)	3.44 (0.04)	.80	3.46 (0.05)	.79	3.46 (0.05)	.16	3.49 (0.04)	.03
Recognition of abuse	3.04 (0.08)	3.01 (0.06)	3.27 (0.05)	.003	3.28 (0.13)	.0052	3.32 (0.03)	.001	3.12 (0.11)	.17
Intentions to intervene	2.56 (0.05)	2.56 (0.08)	2.58 (0.07)	.63	2.46 (0.10)	.25	2.60 (0.08)	.51	2.37 (0.09)	.08
Condom negotiation self-efficacy	3.53 (0.05)	3.47 (0.04)	3.54 (0.06)	.44	3.44 (0.06)	.85	3.51 (0.04)	.89	3.53 (0.06)	.20
Condom and contraceptive attitudes	3.33 (0.03)	3.33 (0.05)	3.31 (0.03)	.40	3.28 (0.04)	.09	3.34 (0.03)	.94	3.38 (0.04)	.15
Positive bystander behaviors, No.^d^ (IQR)	0 (0-1)	0 (0-1)	0 (0-1)	.71	0 (0-1)	.03	0 (0-1)	.36	0 (0-1)	.15
Negative bystander behaviors, No.^d^ (IQR)	1 (0-2)	0 (0-2)	0 (0-2)	.03	0 (0-1)	.69	0 (0-2)	<.001	0 (0-1)	.01

Abbreviations: T2, time point 2 (approximately 3 months after end of program); T3, time point 3 (approximately 9 months after end of program); ARA, adolescent relationship abuse; IQR, interquartile range; NA, not applicable; SV, sexual violence.

^a^ *P* values calculated for within-group differences between baseline and each follow-up time point. Wald log-linear χ^2^ tests were used to calculate *P* values for dichotomous outcomes, and univariate linear regressions were used to calculate *P* values for continuous outcomes. *P* value calculations were restricted to participants who completed follow-up surveys. All testing accounted for clustering.

^b^ SV and ARA items assessed at baseline and T3 only. All clusters were represented in analyses. Standard errors account for neighborhood clustering.

^c^ Among 619 individuals who reported they were dating someone.

^d^ Scale, 0 to 9, indicating number of scenarios with positive or negative responses to each of 9 scenarios witnessed.

**Table 4.** Intervention Effects: Intention-to-Treat Analysis and Post Hoc Adjustment for Intervention Intensity at T2 and T3

Outcome	T2				T3			
	Intervention effect	*P* value	Intensity adjusted	*P* value	Intervention effect, OR (95% CI)	*P* value	Intensity adjusted, OR (95% CI)	*P* value
**Primary outcome**								
								
Any SV or ARA^a^	NA^b^	NA	NA	NA	1.32 (0.86 to 2.01)	.20	1.47 (0.83 to 2.63)	.19
ICC	NA	NA	NA	NA	0.015	NA	0.015	NA
**Additional measures**								
SV and ARA summary score (27 items), RR (95% CI)	NA	NA	NA	NA	1.47 (1.05 to 2.04)	.02	1.71 (1.09 to 2.69)	.02
Any SV (partner or nonpartner)^c^	NA	NA	NA	NA	1.41 (0.78 to 2.53)	.25	1.74 (0.78 to 3.89)	.17
Any ARA^d^	NA	NA	NA	NA	1.12 (0.57 to 2.21)	.75	1.30 (0.51 to 3.29)	.58
Any physical or sexual intimate partner violence^c^^,^^d^	NA	NA	NA	NA	1.38 (0.48 to 3.99)	.55	1.76 (0.41 to 7.49)	.45
Any nonpartner SV^c^	NA	NA	NA	NA	1.55 (0.80 to 3.00)	.20	1.99 (0.80 to 4.91)	.14
Any sexual harassment	NA	NA	NA	NA	1.21 (0.80 to 1.84)	.37	1.27 (0.72 to 2.26)	.41
Any incapacitated sex^c^	NA	NA	NA	NA	1.61 (0.79 to 3.26)	.19	1.83 (0.70 to 4.78)	.22
Incapacitated sex, partner intoxicated^c^	NA	NA	NA	NA	1.41 (0.62 to 3.17)	.41	1.49 (0.50 to 4.51)	.48
Incapacitated sex, provided partner substance^c^	NA	NA	NA	NA	2.13 (0.78 to 5.79)	.14	3.12 (0.80 to 12.18)	.10
Any cyber–sexual abuse	NA	NA	NA	NA	1.71 (1.04 to 2.82)	.04	1.98 (1.00 to 3.93)	.0495
**Secondary outcomes, β (95% CI)**								
Gender-equitable attitudes	−0.01 (−0.08 to 0.06)	.86	0.00 (−0.10 to 0.09)	.95	−0.03 (−0.14 to 0.08)	.60	−0.03 (−0.18 to 0.12)	.73
Recognition of abuse	−0.02 (−0.24 to 0.20)	.85	0.06 (−0.24 to 0.37)	.69	0.18 (−0.05 to 0.41)	.12	0.32 (0.00 to 0.64)	.047
Intentions to intervene	0.10 (−0.12 to 0.31)	.38	0.16 (−0.13 to 0.45)	.28	0.27 (0.03 to 0.52)	.03	0.40 (0.06 to 0.74)	.02
Condom negotiation self-efficacy	0.03 (−0.12 to 0.17)	.72	0.03 (−0.17 to 0.22)	.77	−0.08 (−0.21 to 0.05)	.21	−0.13 (−0.31 to 0.05)	.15
Condom and contraceptive attitudes	0.00 (−0.08 to 0.08)	.93	0.00 (−0.11 to 0.11)	.96	−0.05 (−0.18 to 0.07)	.38	−0.09 (−0.25 to 0.08)	.31
Positive bystander behaviors,^e^ RR (95% CI)	1.4 (0.86 to 2.26)	.17	1.52 (0.79 to 2.93)	.21	1.19 (0.70 to 2.02)	.51	1.28 (0.62 to 2.64)	.50
Negative bystander behaviors,^e^ RR (95% CI)	0.8 (0.48 to 1.33)	.39	0.63 (0.32 to 1.27)	.20	0.95 (0.67 to 1.34)	.75	0.93 (0.58 to 1.50)	.77

Abbreviations: ARA, adolescent relationship abuse; ICC, intraclass correlation coefficient; NA, not applicable; OR, odds ratio; RR, risk ratio; SV, sexual violence; T2, time point 2 (approximately 3 months); T3, time point 3 (approximately 9 months).

^a^ SV and ARA items assessed at baseline and T3 only. All clusters were represented in analyses. All models controlled for race/ethnicity, randomization stratification variable (host site was Young Men’s Christian Association, Urban League, or other), and neighborhood-level clustering. Intensity-adjusted models used a continuous intervention variable indicating the dosage of the intervention received by participants at the level of the round as calculated from attendance and implementer curriculum fidelity. All models with binary SV or ARA outcomes used a binary distribution; models with count-based outcomes (SV and ARA summary score, positive bystander behaviors, and negative bystander behaviors) used a negative binomial distribution. Models with the following continuous outcomes used a gaussian distribution: gender-equitable attitudes, recognition of abuse, intentions to intervene, condom negotiation self-efficacy, and condom and contraceptive attitudes.

^b^ Empty cells for SV and ARA outcomes indicate that these measures were not collected at T2.

^c^ Collapsed race/ethnicity variable was used in these models.

^d^ Among 619 individuals who reported they were dating someone.

^e^ Scale, 0 to 9, indicating number of scenarios with positive or negative responses to each of 9 scenarios witnessed.

Among nonperpetration secondary outcomes, participants in intervention groups reported greater intentions to intervene compared with participants in control groups (β = 0.27; 95% CI, 0.03-0.52; *P* = .03). There were no differences by treatment group in the remaining secondary outcomes.

Prespecified exploratory analyses accounting for intensity of intervention implementation largely reflected primary intention-to-treat results. There was no intervention effect for the primary outcome (OR, 1.47; 95% CI, 0.83-2.63; *P* = .19). Participants in intervention groups were more likely to report recognition of abusive behavior (β = 0.32; 95% CI, 0.00-0.64; *P* = .047) and greater intentions to intervene (β = 0.40; 95% CI, 0.06-0.74; *P* = .02) compared with participants in control groups (Table 4).

An ad hoc analysis of within-arm changes in outcomes over time among participants who completed each follow-up showed significant within-arm differences for the primary outcome and several secondary outcomes (Table 3).

Owing to the association between juvenile justice involvement and completion of the primary outcome at T3, we conducted an additional sensitivity analysis that augmented our primary outcome model with juvenile justice involvement as a covariate. The difference in results was negligible. No adverse events occurred.

## Discussion

This community-based cluster-randomized clinical trial set in low-resource urban neighborhoods compared effectiveness of a gender-transformative curriculum with that of job-readiness training among adolescent boys and young men. We found no significant differences in odds of reporting any SV or ARA perpetration in treatment vs control groups. While receiving the intervention resulted in greater intentions to intervene (and greater recognition of abusive behavior in intervention intensity–adjusted analyses) compared with receiving the control, no significant increases were found in positive bystander behaviors compared with control groups at T3. Contrary to expectations, participants in job-readiness training had greater reductions in reported incidents of SV or ARA perpetration at T3, including cyber–sexual abuse. Participants in job-readiness training also had more equitable gender attitudes at T3, but this difference was not statistically significant between treatment groups.

To our knowledge, this is the first effectiveness study of a gender-transformative program for adolescent boys and young men focused on SV and ARA prevention in the United States. Globally, randomized studies of such interventions have found improvements in gender attitudes and reductions in indicators associated with sexual risk and perpetration of violence against women.^5,8^ Several reasons may exist for differences in results between these international settings and this US study. First, 6 sessions (compared with programs ranging from 14-26 sessions) may not have been adequate for building skills to meaningfully challenge masculinity norms.^8^ Second, the follow-up interval may have been too short. The changes in intentions to intervene are promising, and literature suggests that improvement in bystander attitudes may translate to reduced violence perpetration at the individual level over time.^5,9^ Third, a number of studies in global settings also include a community mobilization component, which, because of funding limitations, was not used in this study. It is possible that involvement of youth in community mobilization would have offered additional opportunities for skills-building.

Fourth, because we analyzed a community-based program set in neighborhoods with concentrated disadvantage, we chose an attention-control design so that all sites would receive an intervention. Job-readiness training, the control, was not expected to have a robust impact on SV or ARA, because of the lack of sexuality, sexual violence, and gender norms content in that training. While such programs are known to reduce youth violence,^14^ they have not been rigorously evaluated for SV or ARA reduction. In this study, job-readiness training had a greater effect on reducing cyber–sexual abuse and promoting more equitable gender attitudes, although differences in the effect on gender attitudes were not significant. We posit that in neighborhoods with concentrated disadvantage, the qualities of the facilitator combined with a dynamic group setting may have contributed to changes in outcomes of interest. Qualitative analyses of observations and interviews are ongoing to describe implementation characteristics. Job-readiness programs may address salient risk and protective factors associated with SV and ARA in these communities, such as economic stress, self-esteem, and future orientation. Although the job-readiness curriculum did not address gender equity, scenarios in the program discussed expected behaviors in the workplace, which may have contributed to reflection on respectful interactions with women. Thus, while this evaluation was not definitive, additional evaluations of Manhood 2.0 and job-readiness training (including on the impact of combining these programs) are warranted.

### Limitations

This study has several limitations, in addition to lacking a true control group. The study was conducted in urban neighborhoods with concentrated disadvantage and so may not generalize to other geographic regions or suburban or rural settings. Participants were adolescent boys and young men who chose to participate in a program about healthy masculinity or job readiness, so they may differ from each other by treatment group on unmeasured factors; furthermore, they may differ from their peers who elected not to participate. All outcomes were self-reported, a known limitation of interpersonal violence research.^1^ Additionally, we did not adjust for prior exposure to violence in our analyses. Because of the anonymous nature of data collection, the dosage of program received (ie, proportion of program completed) could be calculated only at the level of each round rather than at the individual level. Retention of this community-based cohort was also a challenge; individuals at highest risk for school suspension or expulsion were at greater risk for loss to follow-up. Lower-than-anticipated retention may have resulted in an underpowered study. Additionally, participants who were involved in the juvenile justice system (103 individuals [12%]) were difficult to retain because they were fleeing probation or were placed in facilities where study staff were not permitted to follow up. Furthermore, the fact that retention was higher among individuals with more gender-equitable attitudes may reflect selection bias and may have influenced findings. Some prespecified measures (retained per protocol), including gender attitudes and condom use self-efficacy, had lower internal consistency with this sample than reported in a 2019 study.^10^ The intensity-adjusted analysis may have selection bias. Additionally, changes in how much abusive behavior by peers these participants witnessed over time may have affected participants’ opportunities to report positive or negative bystander behaviors. So as to use all available data, not witnessing a behavior was coded as zero; therefore, bystander behaviors may be underestimated.

## Conclusions

This cluster-randomized clinical trial implemented in community settings compared the effectiveness of 2 prevention programs to address SV and ARA perpetration. Further research is needed to assess the effectiveness of gender-transformative programs that combine sexual health education, gender norms change, and bystander skills to reduce SV and ARA perpetration by adolescent boys and young men. In particular, extended time for intervention implementation, supporting youth leadership opportunities to engage in social norms change, community mobilization, and greater attention to practicing skills discussed in the curriculum merit further consideration. While further research is needed, prevention programs that bring adolescent boys and young men together to develop social connections and skills, such as job-readiness skills, may be associated with reducing violence perpetration beyond youth violence. Increasing such opportunities in community settings for adolescent boys and young men, especially those living in neighborhoods with concentrated disadvantage, may be associated with reducing multiple forms of violence and promoting youth flourishing.

## Notes

Supplement 1. **Trial Protocol**

Supplement 2. **eFigure.** Manhood 2.0: Engendering Healthy Masculinities Study Flow **eTable.** Demographic and Secondary Outcome Characteristics Between Study Participants With Complete and Missing Data for the Primary Outcome (Attrition Analysis)

Supplement 3. **Data Sharing Statement**

## References

[1] Smith SG, Zhang X, Basile KC, The National Intimate Partner and Sexual Violence Survey: 2015 Data Brief–Updated Release. National Center for Injury Prevention and Control Centers for Disease Control and Prevention; 2018:1-25.

[2] Dick RN, McCauley HL, Jones KA, Cyber dating abuse among teens using school-based health centers. Pediatrics. 2014;134(6):e1560-e1567. doi:10.1542/peds.2014-0537 25404724

[3] Exner-Cortens D, Eckenrode J, Rothman E Longitudinal associations between teen dating violence victimization and adverse health outcomes. Pediatrics. 2013;131(1):71-78. doi:10.1542/peds.2012-1029 23230075 PMC3529947

[4] World Health Organization Global and Regional Estimates of Violence Against Women: Prevalence and Health Effects of Intimate Partner Violence and Non-Partner Sexual Violence. World Health Organization; 2013.

[5] DeGue S, Valle LA, Holt MK, Massetti GM, Matjasko JL, Tharp AT A systematic review of primary prevention strategies for sexual violence perpetration. Aggress Violent Behav. 2014;19(4):346-362. doi:10.1016/j.avb.2014.05.004 29606897 PMC5875446

[6] Weber AM, Cislaghi B, Meausoone V, ; Gender Equality, Norms and Health Steering Committee Gender norms and health: insights from global survey data. Lancet. 2019;393(10189):2455-2468. doi:10.1016/S0140-6736(19)30765-2 31155273

[7] McCarthy KJ, Mehta R, Haberland NA Gender, power, and violence: a systematic review of measures and their association with male perpetration of IPV. PLoS One. 2018;13(11):e0207091. doi:10.1371/journal.pone.0207091 30496217 PMC6264844

[8] Casey E, Carlson J, Two Bulls S, Yager A Gender transformative approaches to engaging men in gender-based violence prevention: a review and conceptual model. Trauma Violence Abuse. 2018;19(2):231-246. doi:10.1177/1524838016650191 27197533

[9] Lundgren R, Amin A Addressing intimate partner violence and sexual violence among adolescents: emerging evidence of effectiveness. J Adolesc Health. 2015;56(1)(suppl):S42-S50. doi:10.1016/j.jadohealth.2014.08.012 25528978

[10] Kato-Wallace J, Barker G, Garg A, Adapting a global gender-transformative violence prevention program for the U.S. community-based setting for work with young men. Glob Soc Welf. 2019;6(2):121-130. doi:10.1007/s40609-018-00135-y 30956935 PMC6444362

[11] Abebe KZ, Jones KA, Culyba AJ, Engendering healthy masculinities to prevent sexual violence: rationale for and design of the Manhood 2.0 trial. Contemp Clin Trials. 2018;71:18-32. doi:10.1016/j.cct.2018.05.017 29802967 PMC6643273

[12] Nation M, Crusto C, Wandersman A, What works in prevention: principles of effective prevention programs. Am Psychol. 2003;58(6-7):449-456. doi:10.1037/0003-066X.58.6-7.449 12971191

[13] YouthWorks JumpStart Success: Work Readiness and Career Exploration Training. Accessed November 2, 2020. https://www.youthworksinc.org

[14] Heller SB Summer jobs reduce violence among disadvantaged youth. Science. 2014;346(6214):1219-1223. doi:10.1126/science.1257809 25477459

[15] Johnson SL, Jones V, Cheng T. Promoting successful transition to adulthood for urban youths: are risk behaviors associated with career readiness? Soc Work Res. 2014;38(3):144-153.

[16] Ripper L, Ciaravino S, Jones K, Jaime MCD, Miller E Use of a respondent-generated personal code for matching anonymous adolescent surveys in longitudinal studies. J Adolesc Health. 2017;60(6):751-753. doi:10.1016/j.jadohealth.2017.01.003 28279541

[17] Tancredi DJ, Silverman JG, Decker MR, Cluster randomized controlled trial protocol: Addressing Reproductive Coercion in Health Settings (ARCHES). BMC Womens Health. 2015;15:57. doi:10.1186/s12905-015-0216-z 26245752 PMC4527212

[18] Miller E, Tancredi DJ, McCauley HL, “Coaching boys into men”: a cluster-randomized controlled trial of a dating violence prevention program. J Adolesc Health. 2012;51(5):431-438. doi:10.1016/j.jadohealth.2012.01.018 23084163

[19] Espelage DL, Holt MK Dating violence & sexual harassment across the bully-victim continuum among middle and high school students. J Youth Adolesc. 2007;36(6):799-811. doi:10.1007/s10964-006-9109-7

[20] Dartnall E, Jewkes R Sexual violence against women: the scope of the problem. Best Pract Res Clin Obstet Gynaecol. 2013;27(1):3-13. doi:10.1016/j.bpobgyn.2012.08.002 22940107

[21] Koss MP, Gidycz CA Sexual experiences survey: reliability and validity. J Consult Clin Psychol. 1985;53(3):422-423. doi:10.1037/0022-006X.53.3.422 3874219

[22] Pulerwitz J, Barker G Measuring attitudes toward gender norms among young men in Brazil: Development and psychometric evaluation of the GEM scale. Men Masc. 2008;10(3):322-338. doi:10.1177/1097184X06298778

[23] Chu JY, Porche MV, Tolman DL The adolescent masculinity ideology in relationships scale: development and validation of a new measure for boys. Men Masc. 2005;8(1):93-115. doi:10.1177/1097184X03257453

[24] Rothman EF, Decker MR, Silverman JG Evaluation of a teen dating violence social marketing campaign: lessons learned when the null hypothesis was accepted. New Dir Eval. 2006;2006(110):33-44. doi:10.1002/ev.185

[25] Brafford LJ, Beck KH Development and validation of a condom self-efficacy scale for college students. J Am Coll Health. 1991;39(5):219-225. doi:10.1080/07448481.1991.9936238 1783705

[26] Cabral RJ, Galavotti C, Stark MJ, Psychosocial factors associated with stage of change for contraceptive use among women at increased risk for HIV and STDs. J Appl Soc Psychol. 2004;34(5):959-983. doi:10.1111/j.1559-1816.2004.tb02579.x

[27] Aalsma MC, Carpentier MY, Azzouz F, Fortenberry JD Longitudinal effects of health-harming and health-protective behaviors within adolescent romantic dyads. Soc Sci Med. 2012;74(9):1444-1451. doi:10.1016/j.socscimed.2012.01.014 22424832 PMC3405728

[28] Borrero S, Farkas A, Dehlendorf C, Rocca CH Racial and ethnic differences in men’s knowledge and attitudes about contraception. Contraception. 2013;88(4):532-538. doi:10.1016/j.contraception.2013.04.002 23697702 PMC3758769

[29] Carvajal DN, Ghazarian SR, Shea Crowne S, Is depression associated with contraceptive motivations, intentions, and use among a sample of low-income Latinas? Womens Health Issues. 2014;24(1):e105-e113. doi:10.1016/j.whi.2013.10.003 24439935

[30] Hemming K, Girling AJ, Sitch AJ, Marsh J, Lilford RJ Sample size calculations for cluster randomised controlled trials with a fixed number of clusters. BMC Med Res Methodol. 2011;11:102. doi:10.1186/1471-2288-11-102 21718530 PMC3149598

[31] Miller E, Tancredi DJ, McCauley HL, One-year follow-up of a coach-delivered dating violence prevention program: a cluster randomized controlled trial. Am J Prev Med. 2013;45(1):108-112. doi:10.1016/j.amepre.2013.03.007 23790995

