# Supplementary material for: Effect of a Community-Based Gender Norms Program on Sexual Violence Perpetration by Adolescent Boys and Young Men: A Cluster Randomized Clinical Trial
Source: JAMA Netw Open. 2020 Dec 22;3(12):e2028499. doi: 10.1001/jamanetworkopen.2020.28499 (PMC7756236; doi:10.1001/jamanetworkopen.2020.28499)
Supplement: Supplement 1. — Trial Protocol [file jamanetwopen-e2028499-s001.pdf]

TITLE: Engendering Healthy Masculinities to Prevent Sexual Violence:

Cluster-randomized trial of Manhood 2.0 a community-based gender norms program for adolescent males

Protocol Number: PRO14080673

National Clinical Trial (NCT) Identified Number: NCT02427061

Principal Investigator: Elizabeth Miller

Sponsor: University of Pittsburgh

Grant Title: Engendering Healthy Masculinities to Prevent Sexual Violence

Grant Number*: U01CE002528

Funded by: Centers for Disease Control and Prevention

Version Number: v.28

July 18, 2018

CONFIDENTIALITY STATEMENT

This document is confidential communication. Acceptance of this document constitutes agreement by the recipient that no unpublished information contained herein will be published or disclosed without prior approval of the Principal Investigator or other participating study leadership and as consistent with the CDC terms of award.

Table of Contents

STATEMENT OF COMPLIANCE 1
INVESTIGATOR'S SIGNATURE 2
1 PROTOCOL SUMMARY 3
1.1 Synopsis 3
1.2 Schema 4
1.3 Schedule of Activities 4
2 INTRODUCTION 5
2.1 Study Rationale 5
2.2 Background 5
2.3 Risk/Benefit Assessment 6
2.3.1 Known Potential Risks 6
2.3.2 Known Potential Benefits 6
2.3.3 Assessment of Potential Risks and Benefits 6
3 OBJECTIVES AND ENDPOINTS 6
4 STUDY DESIGN 8
4.1 Overall Design 8
4.2 Scientific Rationale for Study Design 9
4.3 Justification for Intervention 9
4.4 End-of-Study Definition 10
5 STUDY POPULATION 11
5.1 Inclusion Criteria 11
5.2 Exclusion Criteria 11
5.3 Lifestyle Considerations 11
5.4 Screen Failures 11
5.5 Strategies for Recruitment and Retention 11
6 STUDY INTERVENTION(S) OR EXPERIMENTAL MANIPULATION(S) 13
6.1 Study Intervention(s) or Experimental Manipulation(s) Administration 13
6.1.1 Study Intervention or Experimental Manipulation Description 13
6.1.2 Administration and/or Dosing 13
6.2 Fidelity 14
6.2.1 Interventionist Training and Tracking 14
6.3 Measures to Minimize Bias: Randomization and Blinding 15
6.4 Study Intervention/Experimental Manipulation Adherence 16
6.5 Concomitant Therapy 16
6.5.1 Rescue Therapy 16
7 STUDY INTERVENTION/EXPERIMENTAL MANIPULATION DISCONTINUATION AND PARTICIPANT DISCONTINUATION/WITHDRAWAL 16
7.1 Discontinuation of Study Intervention/Experimental Manipulation 16
7.2 Participant Discontinuation/Withdrawal from the Study 16
7.3 Lost to Follow-Up 17
8 STUDY ASSESSMENTS AND PROCEDURES 17
8.1 Endpoint and Other Non-Safety Assessments 17
8.2 Safety Assessments 21
8.3 Adverse Events and Serious Adverse Events 23
8.3.1 Definition of Adverse Events 23
8.3.2 Definition of Serious Adverse Events 23

8.3.3 Classification of an Adverse Event ... 23
8.3.4 Time Period and Frequency for Event Assessment and Follow-Up ... 24
8.3.5 Adverse Event Reporting ... 25
8.3.6 Serious Adverse Event Reporting ... 25
8.3.7 Reporting Events to Participants ... 25
8.3.8 Events of Special Interest ... 25
8.3.9 Reporting of Pregnancy ... 25
8.4 Unanticipated Problems ... 25
8.4.1 Definition of Unanticipated Problems ... 25
8.4.2 Unanticipated Problems Reporting ... 26
8.4.3 Reporting Unanticipated Problems to Participants ... 26
9 STATISTICAL CONSIDERATIONS ... 26
9.1 Statistical Hypotheses ... 26
9.2 Sample Size Determination ... 27
9.3 Populations for Analyses ... 27
9.4 Statistical Analyses ... 27
9.4.1 General Approach ... 27
9.4.2 Analysis of the Primary Endpoint(s) ... 28
9.4.3 Analysis of the Secondary Endpoint(s) ... 28
9.4.4 Safety Analyses ... 28
9.4.5 Baseline Descriptive Statistics ... 29
9.4.6 Planned Interim Analyses ... 29
9.4.7 Sub-Group Analyses ... 29
9.4.8 Tabulation of Individual Participant Data ... 29
9.4.9 Exploratory Analyses ... 29
10 SUPPORTING DOCUMENTATION AND OPERATIONAL CONSIDERATIONS ... 30
10.1 Regulatory, Ethical, and Study Oversight Considerations ... 30
10.1.1 Informed Consent Process ... 30
10.1.2 Study Discontinuation and Closure ... 30
10.1.3 Confidentiality and Privacy ... 30
10.1.4 Future Use of Stored Specimens and Data ... 31
10.1.5 Key Roles and Study Governance ... 32
10.1.6 Safety Oversight ... 32
10.1.7 Clinical Monitoring ... 32
10.1.8 Quality Assurance and Quality Control ... 33
10.1.9 Data Handling and Record Keeping ... 33
10.1.10 Protocol Deviations ... 34
10.1.11 Publication and Data Sharing Policy ... 34
10.1.12 Conflict of Interest Policy ... 34
10.2 Additional Considerations ... 35
10.3 Abbreviations and Special Terms ... 35
10.4 Protocol Amendment History ... 36
11 REFERENCES ... 40

STATEMENT OF COMPLIANCE

The trial will be carried out in accordance with International Council on Harmonisation Good Clinical Practice (ICH GCP) and the following:

- United States (US) Code of Federal Regulations (CFR) applicable to clinical studies (45 CFR Part 46, 21 CFR Part 50, 21 CFR Part 56, 21 CFR Part 312, and/or 21 CFR Part 812).

All personnel involved in the conduct of this study have completed Human Subjects Protection and ICH GCP Training.

The protocol, informed consent form(s), recruitment materials, and all participant materials will be submitted to the IRB for review and approval. Approval of both the protocol and the consent form(s) must be obtained before any participant is consented. Any amendment to the protocol will require review and approval by the IRB before the changes are implemented to the study. All changes to the consent form(s) will be IRB approved; a determination will be made regarding whether a new consent needs to be obtained from participants who provided consent, using a previously approved consent form.

INVESTIGATOR'S SIGNATURE

The signature below constitutes the approval of this protocol and provides the necessary assurances that this study will be conducted according to all stipulations of the protocol, including all statements regarding confidentiality, and according to local legal and regulatory requirements and applicable US federal regulations and ICH guidelines, as described in the *Statement of Compliance* above.

Principal Investigator or Clinical Site Investigator:

Signed: ______________________ Date: 12/23/2014

Name: Elizabeth Miller

Title: Director, Adolescent and Young Adult Medicine

Investigator Contact Information
Affiliation: University of Pittsburgh School of Medicine, Department of Pediatrics
Address: 120 Lytton Ave., Suite 302, Pittsburgh, PA 15213
Telephone: 412-692-8504
Email: elizabeth.miller@chp.edu

1 PROTOCOL SUMMARY

1.1 SYNOPSIS

**Title:**	Engendering Healthy Masculinities to Prevent Sexual Violence
**Grant Number:**	U01CE002528
**Study Description:**	Adapted from an international setting for use in the US, "Manhood 2.0" is a "gender transformative" program that involves challenging harmful gender and sexuality norms that foster violence against women while promoting bystander intervention (*i.e.*, giving boys skills to interrupt abusive behaviors they witness among peers) to reduce the perpetration of sexual violence (SV) and adolescent relationship abuse (ARA). Manhood 2.0 is being rigorously evaluated in a community-based cluster-randomized trial in 21 lower resource Pittsburgh neighborhoods with 866 adolescent males ages 13-19. The comparison intervention is a job readiness training program which focuses on the skills needed to prepare youth for entering the workforce, including goal setting, accountability, resume building, and interview preparation. This study will provide urgently needed information about the effectiveness of a gender transformative program, which combines healthy sexuality education, gender norms change, and bystander skills to interrupt peers' disrespectful and harmful behaviors to reduce SV/ARA perpetration among adolescent males.
**Objectives:**	The primary objective of this study is to test the effectiveness of Manhood 2.0 compared to a job skills curriculum on 1) reductions in self-reported perpetration of SV and ARA (*Primary Outcome)* toward females and 2) increased positive bystander intervention behaviors (*Secondary outcome*).
**Endpoints:**	Primary Endpoint: change in participant-level perpetration of sexual violence or adolescent relationship abuse (intention-to-treat analysis) at T3<br>Secondary Endpoints: change in positive bystander behavior from baseline to follow up<br>Additional Endpoints: change in intentions to intervene from baseline to follow up; change in recognition of abuse from baseline to follow up; change in gender equitable attitudes from baseline to follow up; change in condom use self-efficacy from baseline to follow up; change in contraception attitudes from baseline to follow up
**Study Population:**	Adolescent males (13-19 years old) living in 20 Pittsburgh, Pennsylvania neighborhoods with concentrated disadvantage.
**Phase or Stage:**	Phase 3 cluster-randomized controlled trial
**Description of Sites/Facilities Enrolling Participants:**	Within the 20 participating neighborhoods, sites include places of worship, community centers, public libraries, and juvenile justice community intensive surveillance program centers.

**Description of Study Intervention/Experimental Manipulation:**	This study design involves a two-arm cluster-randomized-controlled trial conducted with adolescent males ages 13-19 recruited from youth-serving community agencies in Pittsburgh, PA. Twenty-one clusters from 20 neighborhoods are randomly allocated to the intervention or control arm. Both experimental and control arm interventions involve 18 hours of curriculum, generally spread out over 3 to 6 week periods. The program is delivered with some variation in schedules to meet the needs of community partners and participating youth.<br>Intervention Arm: Manhood 2.0 curriculum. Youth are guided to explore social constructions of masculinity, describe healthy relationships, discuss healthy sexual behaviors, identify coercive and disrespectful behaviors, and practice skills to intervene when witnessing peers' disrespectful and harmful behaviors, with repeated reflection on gender norms throughout these sessions<br>Control Arm: Job readiness training curriculum
**Study Duration:**	Study start April 2015 – estimated 3 years to complete data collection
**Participant Duration:**	1 year

1.2 SCHEMA

Figure 1: Manhood 2.0: Engendering Healthy Masculinities -- Study Flow

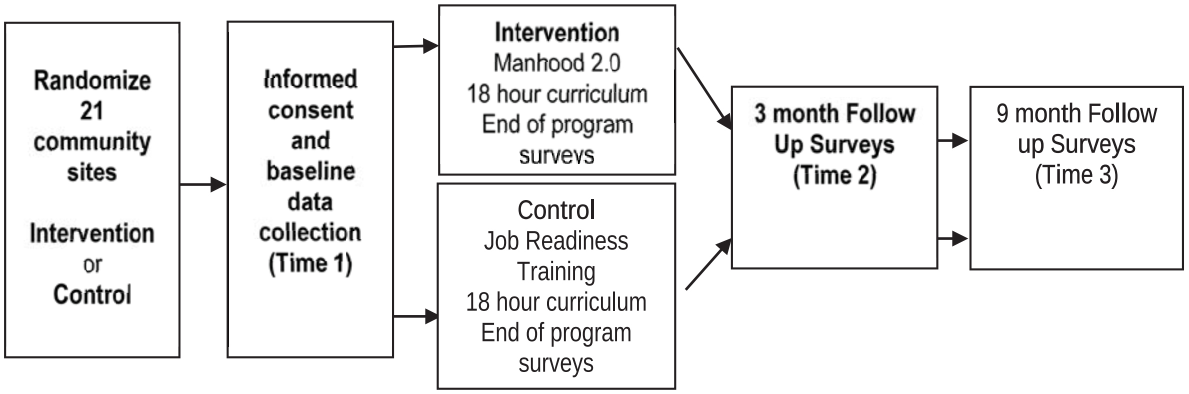

1.3 SCHEDULE OF ACTIVITIES

	Entry into program	End of Program About 6 weeks	Time 2 - About 3 months from EOP	Time 3- About 9 months from EOP	Interview after surveys
Informed Consent	X				

Control & Experimental Interventions – Occupational therapy	X	X			
Adverse Events Reporting	X	X	X	X	X
**Outcome Evaluation**					
Baseline Survey	X				
Follow up Surveys		X	X	X	
Interview					X

2 INTRODUCTION

2.1 STUDY RATIONALE

This study aims to test, via a two arm cluster randomized controlled trial, a "gender transformative" sexual violence (SV)/adolescent relationship abuse (ARA) perpetration prevention program among African American adolescent males ages 13-19 implemented in a community-based setting titled "Manhood 2.0." "Gender transformative" refers to a theory- and evidence-based approach to alter gender norms that foster SV/ARA while promoting bystander intervention (i.e., giving boys skills to interrupt abusive behaviors they witness among peers) to reduce SV/ARA perpetration. As the acceptance of SV and involvement in unhealthy sexual behaviors are associated with SV/ARA perpetration, this program integrates analysis of social norms that condone violence against women, sexual health promotion, and skills in bystander intervention -- an approach that has been implemented in multiple non-U.S. settings among young adult males with reductions in violence, development of more equitable gender attitudes, and less risky sexual behaviors.

2.2 BACKGROUND

Sexual violence (SV) and intimate partner violence affect at least one in three women in the world (1) including in the United States.(2) Among adolescents in the US, non-partner SV often co-occurs with adolescent relationship abuse (ARA; physical, sexual, or emotional abuse by a partner) victimization,(3) and such experiences are associated with poor health, including suicidality, depression, substance use, unintended pregnancy, and sexually transmitted infections (STIs).(4–13) Perpetration of SV/ARA is associated with multiple individual and contextual factors, including exposure to adverse childhood experiences, poor conflict resolution and relationship skills, and norms that condone violence perpetration.(14) Prevention entails modifying potential perpetrator behaviors, which in turn requires attention to both individual attitudes and the normative peer context. (14)

As SV/ARA perpetration often emerges in the context of male peers who demonstrate negative attitudes toward females, endorse bias-based prejudices regarding homosexuality and condone abuse perpetration,(15–22) prevention requires addressing potential perpetrator attitudes and behaviors as well as the gendered peer environment in which they are embedded. Perceived peer tolerance for SV/ARA may promote individual likelihood of these behaviors, and may reduce comfort and ability to intervene when faced with negative behaviors among peers, contributing to a social climate that enables such behavior.(17) Many violence prevention programs focused on social norms change employ a bystander behavior approach, in which individuals are taught skills to respond with active intervention in SV/ARA rather than with apathy or tolerance.(23,24). This study draws on building bystander intervention skills

combined with evidence from international settings that demonstrated the effectiveness of encouraging critical analysis of gender norms, challenging homophobia and gender-based harassment, and building skills both to critically question harmful masculine norms and to employ more equitable behaviors.

The literature on adolescent sexual health promotion also underscores the need for skills building that includes an emphasis on respect, communication about pregnancy and STI and HIV/AIDS prevention, condom negotiation, sexual consent, and learning about reproductive and sexual coercion.(25–27) The most effective sexual health interventions also address gender and power.(28) Integration of open, in-depth discussions about respectful sexual behaviors that also address homophobia and rigid masculinity norms may simultaneously reduce SV/ARA perpetration and improve sexual health. In international settings, sexual health promotion programs that incorporate changing cultural norms around masculinity (i.e., "gender transformative" programs), focused on older adolescents and young adults, have demonstrated significant positive shifts in gender attitudes as well as increased use of condoms and decreased reporting of men's use of violence towards an intimate partner.(18,29–32) This is the first study to test the effectiveness of a community-based program for adolescent males that combines healthy sexuality skills, gender norms change, and bystander skills to interrupt peers' disrespectful and harmful behaviors to prevent SV/ARA perpetration among adolescent males.

2.3 RISK/BENEFIT ASSESSMENT

2.3.1 KNOWN POTENTIAL RISKS

This evaluation itself involving anonymous surveys and interviews is of minimal risk to participants. The risks are potential breach of confidentiality, and the potential that some of the questions asked may be too sensitive or emotionally distressing for participants.

2.3.2 KNOWN POTENTIAL BENEFITS

There is no direct benefit to research participants.

2.3.3 ASSESSMENT OF POTENTIAL RISKS AND BENEFITS

This study is of minimal risk to participants. It is possible that some youth may find some of the survey questions too sensitive or uncomfortable. Participation is voluntary; participants are reminded that they can stop participating at any point. Youth are provided with relevant resources at the end of each survey. The youth surveys are anonymous. That is, youth produce a self-generated personal code based on 8 non-identifiable questions (e.g., first letter of mother's first name) to ensure responses will remain as anonymous as possible and can be matched across data collection points. No names or linking information will be connected to the surveys.

3 OBJECTIVES AND ENDPOINTS

OBJECTIVES	ENDPOINTS	JUSTIFICATION FOR ENDPOINTS
Primary		
Reduction of sexual violence and adolescent relationship abuse	Change in self-reported perpetration of Sexual Violence and Adolescent Relationship Abuse at Time 3 --<br>Assessment of Sexual Violence and Adolescent Relationship Abuse perpetration comparing baseline summary score with follow up summary score	Primary goal of sexual violence prevention programming
Secondary		
Improvement in positive bystander behaviors (intervening when witnessing peers' disrespectful and harmful behaviors)	Change in Positive Bystander Behavior from Baseline to Follow Up -- Assessment of positive bystander behavior in participants over the past 3 months when witnessing disrespectful and harmful behavior among peers comparing baseline and follow up summary scores. Participants report if they have witnessed peers' abusive behaviors in the past 3 months and if witnessed, how they responded (whether they intervened to interrupt the behavior)	Mediator - increasing skills in recognizing and intervening in peers' disrespectful behaviors are expected to reduce individuals' use of violence in their own relationships
Tertiary/Exploratory		
Additional Endpoints:<br>Intentions to intervene<br><br>Recognition of Abuse	1. change in intentions to intervene from baseline to follow up -- Proclivity to intervene when witnessing disrespectful and harmful behaviors among peers comparing baseline and follow up mean scores on a scale assessing likelihood of trying to stop disrespectful behaviors among peers<br>2. change in recognition of abuse from baseline to follow up -- Recognition of disrespectful and harmful	1-3 Mediators<br><br>4-5 exploratory outcomes of program on sexual health behaviors

OBJECTIVES	ENDPOINTS	JUSTIFICATION FOR ENDPOINTS
	behaviors against girls as abusive comparing baseline and follow up mean scores on the recognition of abusive behavior scale<br>3. change in gender equitable attitudes from baseline to follow up -- Assessment of gender-equitable attitudes comparing baseline and follow up mean scores on gender attitudes scale<br>4. change in condom use self-efficacy from baseline to follow up - Assessment of self-efficacy to use condoms comparing baseline and follow up mean scores on a scale assessing confidence to negotiate condom use<br>5. change in contraception attitudes from baseline to follow up -- Assessment of attitudes related to contraceptive use comparing baseline and follow up mean scores on a scale assessing contraception attitudes	

4 STUDY DESIGN

4.1 OVERALL DESIGN

This study design involves a two-arm cluster-randomized-controlled trial conducted with adolescent males ages 13-19 recruited from youth-serving community agencies in Pittsburgh, PA. Twenty-one clusters from 20 neighborhoods were randomly allocated to the intervention or control arm. Participants (n=866) complete surveys prior to program implementation (baseline) and immediately following the program (end of program, EOP). Follow-up surveys are collected 3 months (T2) and 9 months (T3) after end of program. Baseline surveys are completed in-person using tablets to complete the survey online; EOP, T2, and T3 are also completed in-person on a tablet or remotely using survey links that are texted or emailed to participants using contact information provided with recruitment. Retention is facilitated by collecting detailed contact information and offering incentives for survey completion.

Randomization was performed at the neighborhood level (i.e., cluster) to reduce risk for contamination. The initial randomization included 10 clusters that were assigned to experimental or control conditions. The study statistician performed this randomization, stratifying by lead site in that neighborhood (YMCA, Urban League, or Other), such that within each stratum, each site/neighborhood had a 50/50 chance of being assigned to intervention or control. Due to a combination of lower-than-expected recruitment by neighborhood (target=96 participants each) and the interest from other community partners, we individually randomized an additional 11 neighborhoods, stratified by type of site. This resulted in a total of 21 clusters randomized, with 11 assigned to experimental and 10 assigned to control. All neighborhoods in the study met the original criteria of being socially or economically disadvantaged and/or predominantly African American.

Aim 1: To test, via a 2-arm cluster-randomized trial in youth-serving agencies, the effectiveness of "Manhood 2.0," an 18 hour SV/ARA prevention program compared to an 18 hour life skills development curriculum. Approximately 3 months after end of program implementation), compared to controls, youth will demonstrate increased positive bystander intervention behaviors (Secondary outcome, H1.1). Intermediate outcomes are: increased condom and contraceptive self-efficacy (H1.2); increased recognition of abusive behaviors (H1.3); increased gender-equitable attitudes (H1.4); and increased intentions to intervene with peers (H1.5).
Aim 2: To examine whether "Manhood 2.0" results in sustained improvements in these same Aim 1 outcomes assessed at approximately 9 months after end of program implementation (Time 3), as well as reductions in self-reported perpetration of SV and ARA (Primary Outcome) toward females from baseline to Time 3, compared to controls.
Aim 3: To explore whether demographics, participants' pre-intervention risk and protective factors (e.g., history of SV/ARA exposure, sexual risk, connectedness), and setting-level characteristics (e.g., staff experience, organizational capacity) are associated with differences in outcomes.

4.2 SCIENTIFIC RATIONALE FOR STUDY DESIGN

This design builds on primary prevention principles that emphasize a comprehensive, theory-driven approach, sociocultural relevance, well-trained staff, opportunity for building positive relationships with youth, sufficient dosage (through repeated exposure to content), and youth participation balanced with feasibility and cost of implementation approaches.

Randomization was performed at the neighborhood level (i.e., cluster) to reduce risk for contamination.

4.3 JUSTIFICATION FOR INTERVENTION

Sexual violence (SV) and intimate partner violence affect at least one in three women in the world including in the United States. Among adolescents in the US, non-partner SV often co-occurs with adolescent relationship abuse (ARA; physical, sexual, or emotional abuse by a partner) victimization, and such experiences are associated with poor health, including suicidality, depression, substance use, unintended pregnancy, and sexually transmitted infections (STIs). Perpetration of SV/ARA is associated with multiple individual and contextual factors, including exposure to adverse childhood experiences, poor conflict resolution and relationship skills, and norms that condone violence perpetration. Prevention entails modifying potential perpetrator behaviors, which in turn requires attention to both individual attitudes and the normative peer context.

To best address the multiple factors that influence violence, approaches much achieve several aims. First, A program that integrates gender norms change, education about healthy sexuality, and promotion of positive bystander intervention behaviors is likely to address several modifiable risk factors related to

SV/ARA perpetration. Community-based SV/ARA prevention programs which are flexible around how and when curriculum is delivered and which do not rely on school district approvals for implementation are needed. Youth development-focused community programs engage adolescent males living in socially disadvantaged neighborhoods through a range of modalities including community-based athletics, after school programs, employment programs, and truancy programs. Finally, sustainable, scalable community programs to prevent SV are needed.

We have too few evidence-based SV/ARA prevention programs in the U.S. focused on adolescents that can be delivered by community members/youth agency staff without extensive training. We have only one evidence-based SV/ARA prevention program focused on adolescent males and that is in the context of school-based athletics only. No evidence-based SV/ARA programs for youth take place outside of the classroom or school-based athletics setting. This study will advance scientific knowledge about SV/ARA perpetration prevention (with an emphasis on primary prevention) and address these gaps in the existing evidence base. This research will provide urgently needed information about the relevance of an innovative community-based SV/ARA prevention program adapted from international prevention efforts for implementation with adolescent males in community-based settings.

The intervention involves an 18 hour curriculum divided into six 3 hour sessions delivered once or twice a week (generally over 3 to 6 week time period). This design builds on primary prevention principles that emphasize a comprehensive, theory-driven approach, sociocultural relevance, well-trained staff, opportunity for building positive relationships with youth, sufficient dosage (through repeated exposure to content), and youth participation balanced with feasibility and cost of implementation approaches. Program implementation relies on the youth development infrastructure and community-based networks already in place at participating YMCAs, Urban League, and other youth development organizations in Pittsburgh, including the ability to reach diverse adolescent males with the assistance of other youth-serving community agencies including schools, libraries, and churches. Multiple stakeholders at the local, regional, national, and global level are involved to ensure the program is relevant, easy to implement, and replicable, thus if found to be effective, could be widely disseminated as a promising prevention program.

Power and sample size calculations were based on clinically meaningful differences between treatment groups with respect to changes in the outcomes across time (i.e. intervention effect). For the primary outcome - any SV/ARA perpetration at Time 3, the detectable difference between arms was calculated based on traditional methods that assumed a fixed number of clusters as well as fixed number of subjects per cluster. Twenty-one clusters were randomized, assuming a within-cluster intra-class correlation (ICC) of 0.01 (within-school correlations for abuse perpetration, similar to our team's prior work with a related sexual violence prevention program with male athletes), and a 20% baseline SV perpetration rate in the control arm. With 866 participants (approximately 41 boys at each site) and an 80% retention rate at Time 3, we expect to have 80% power to detect an 8.3 point decrease in SV perpetration (42% relative decrease) due to the intervention. We anticipate having ample power to detect clinically meaningful changes in bystander behaviors and the secondary outcomes as well. Based on previous studies, the within-cluster ICCs for each of our secondary outcomes ranged from 0.006 to 0.01. If we assume the upper end of that range, we will have at least 80% power to detect standardized mean differences as small as 0.23 between study arms.

4.4 END-OF-STUDY DEFINITION

A participant is considered to have completed the study if they complete (1) the baseline assessment, (2) attend either the intervention program, an 18 hour curriculum delivered over multiple sessions, typically between a 3 week to 2 month period, or the control program, an 18 hour evidence-based job skills

curriculum, (3) complete an end of program survey, (4) complete a Time 3 survey approximately nine months after the completing either program. Participants may also complete a Time 2 survey approximately three months after the end of the program.

End of study is defined as completion of T3 survey data collection and an optional interview about their experience in the program.

5 STUDY POPULATION

5.1 INCLUSION CRITERIA

Neighborhood Eligibility: Neighborhoods were recruited by identifying a potential community partner who could host the program and were willing to be randomized to receive the intervention or control programming. Neighborhoods were identified based on having sites where the YMCA, Urban League, or other youth serving partners had existing programs and which were considered lower income communities based on census information and school district data.

Youth Eligibility:
Eligible youth are between the ages of 13 to 19, who identify themselves as male, are residents in the neighborhood where the site is located, and willing to participate in an 18 hour gender-specific program or job readiness training (programs already determined based on neighborhood). Youth must verbally assent into the research.

5.2 EXCLUSION CRITERIA

Youth are excluded if planning on moving away from the Pittsburgh area within 3 months

5.3 LIFESTYLE CONSIDERATIONS

N/A

5.4 SCREEN FAILURES

Participants who assent in the program but do not meet eligibility criteria are invited to return when they meet criteria. Examples include interested youth who are not yet 13 years old.

5.5 STRATEGIES FOR RECRUITMENT AND RETENTION

Study setting

This study involves 20 neighborhoods and 21 clusters in the Pittsburgh area; within each neighborhood, one to four different community partner organizations (referred to as “community

partners" here) participated. Neighborhoods were recruited by identifying a potential community partner who could host the program and were willing to be randomized to receive the intervention or control programming. These community partners included youth-serving organizations, YMCA, Urban League, faith-based organizations, and libraries. Additionally, we partnered with the county's community intensive surveillance program, a diversion program for youth involved in the juvenile justice system.

Neighborhoods were identified based on having sites where the YMCA, Urban League, or other youth serving partners had existing programs and which were considered lower income communities based on census information and school district data. Asset maps were created for each neighborhood with the goal of identifying community champions and youth-relevant resources to support this community-based project. The strong partnerships established with key stakeholders in each of these sites (including site coordinators, facilitators, and community members) facilitated recruitment and retention as described below.

Among the 20 participating neighborhoods, the proportion of students attending public high schools in those neighborhoods considered economically disadvantaged ranges from 32-100%; the high school graduation rate for those same school districts ranged from 63-97%. Each of these neighborhoods struggle with poverty, school 'push-out' (disciplinary actions that push youth, especially African American boys, out of the regular school system), and among the highest rates of gang and gun violence in the county (see Table 2 for neighborhood characteristics). These characteristics were compared by treatment arm using Kolmogorov-Smirnov two-sample exact tests due to their non-normal distributions. Neighborhood characteristics did not vary between the intervention and control arm neighborhoods.

Within the 20 participating neighborhoods, there were a total of 40 sites/community partners/locations approached and 38 agreed to participate. The composition of the participating sites included 11 places of worship, 20 community centers, 2 public libraries, and 5 juvenile justice community intensive surveillance program centers. One site (cluster) was located at the downtown Urban League office and consisted of youth involved in an African American young men's leadership group who came from several different neighborhoods and schools in the Pittsburgh area.

For recruitment of eligible youth, we rely on this network of community partners identified in asset maps created for each neighborhood cluster. This includes site leaders, program facilitators, recruiters with strong connections to their community, prevention specialists embedded in schools, school districts that offer community-based programs as an alternative to suspension, and the Community Intensive Supervision Program (CISP) for youth involved in the juvenile justice system.

Using respondent driven sampling (RDS), former and current participants may refer their friends to the program. Participants interested in RDS receive a packet of information on how to recruit a friend or neighbor and five recruitment coupons. When newly-recruited participants come to their first session and turn in their recruitment coupon, the peer recruiter is compensated $5, up to $25 overall.

Once community partners recruit youth, retention throughout the 18-hour curriculum is a key focus. Upon enrolling in either the control or experimental arm, prospective participants receive program information, sign an assent form and complete a contact information sheet. Research assistants ensure that the document is legible and that all fields are completed. Research assistants use this information to contact the participant prior to each session to remind them of the session. If youth are not present at the beginning of a session, research assistants and facilitators will contact the participant to encourage them to join late.

Justification <18 Year Olds:

Evidence that sexual violence (SV), adolescent relationship abuse (ARA) and gender-based harassment behaviors are prevalent in adolescence, and increase as youth age strongly supports the need to interrupt the emergence of gender inequitable practice and associated SV/ARA perpetration in adolescence in the context of emerging sexuality. While SV prevention programs are clearly needed for middle school youth, as most middle school youth are not yet engaged in sexual relationships, integrating education about reproductive and sexual decision making (i.e., consent, condom negotiation skills, pregnancy and

infection prevention communication) is most developmentally appropriate for youth ages 14 to 17 (target age 14-17, may include up to age 19 and as young as 13). Study is minimal risk.

Justification for Prisoners:
Conduct follow up surveys with already enrolled participants who become incarcerated during the course of the project period. The surveys are confidential and voluntary, and designed to be anonymous, as each participant creates their own identification code that only they know the answer to. The primary risks associated with participation are potential breach of confidentiality and emotional distress associated with answering some of the questions.

Youth will receive up to $50 for research activities conducted over the 18 hours of curriculum. Increment amounts will be decided upon by the site lead and PI for each neighborhood and will be based on how best to serve the youth in each area. Youth will receive $30 for the first follow up survey; and $50 for the second (and final) follow up survey. Youth participating in the end of study interview will receive $30. Subjects who become incarcerated during the follow up period will not receive monetary compensation until they are released and have completed the study procedures.

6 STUDY INTERVENTION(S) OR EXPERIMENTAL MANIPULATION(S)

6.1 STUDY INTERVENTION(S) OR EXPERIMENTAL MANIPULATION(S) ADMINISTRATION.

6.1.1 STUDY INTERVENTION OR EXPERIMENTAL MANIPULATION DESCRIPTION

Experimental and Control Arms:
Program Delivery: Both experimental and control arm interventions involve 18 hours of curriculum, generally spread out over 3 to 6 week periods. The program is delivered with some variation in schedules to meet the needs of community partners and participating youth. Such configurations include but are not limited to: three 6-hour sessions spanning three weekdays during the summer (for job skills training only), nine 2-hour sessions held twice per week on weekday evenings, and six 3-hour sessions held once or twice per week on weekday evenings or Saturday afternoons (which was the preferred and most commonly used design).
Fidelity to intervention: For both intervention and control arms, research assistants are present at every session to track attendance, facilitate program implementation logistics (such as ordering food), and to complete detailed fidelity forms that assess facilitators' ability to deliver the content of the program as intended.
Manhood 2.0 (experimental arm): Manhood 2.0 guides youth to explore and reflect upon social constructions of masculinity, describe healthy relationships, discuss healthy sexual behaviors, identify coercive and disrespectful behaviors, and practice skills to intervene when witnessing peers' disrespectful and harmful behaviors, with repeated reflection on gender norms throughout these sessions. The curriculum involved three main topic areas. The first focuses on the theme of gender, masculinity and power, allowing the young men to actively reflect on the messages and expectations that they have received from society about manhood and gender norms. The second topic focuses on the theme of violence. This includes several components: an exploration of the various forms of violence, its impact on communities, and the role that masculinity may contribute; identifying healthy versus unhealthy romantic relationships; sexual consent and decision making; and bystander interventions when witnessing abusive behavior. The final topic area focuses on sexual and reproductive health, which includes providing

information about sexual health and contraception, condom and contraceptive demonstrations, tying health behaviors and access to health facilities to conceptions of masculinity, and opportunities to ask medical professionals sexual health-related questions.

Job Skills Curriculum (control arm): The job skills readiness program was developed and tested, and is widely used throughout the county, called "Jump Start Success Work Readiness and Career Exploration Training" (*http://www.youthworksinc.org/jumpstart_success/index.html*). The sessions were set up to mimic the timing for the intervention curriculum (18 hour curriculum).
Program Content: The curriculum covers topics from career options and goal setting to interviewing skills. The entire curriculum involves 9 modules. For the purposes of this study, the facilitators focused on the first 6 modules (to mimic Manhood 2.0 structure) with an emphasis on goal setting, future orientation, learning about building a resume, interviewing skills, and workplace expectations.

6.1.2 ADMINISTRATION AND/OR DOSING

Tracking Dosage: Attendance forms are completed at each site for each round. As surveys are anonymous, tracking number of sessions for each individual is not feasible. Thus, dosage will be calculated for each round based on % overall attendance.

6.2 FIDELITY

6.2.1 INTERVENTIONIST TRAINING AND TRACKING

Training of Facilitators for Control and Intervention:

The initial training for Manhood 2.0 occurred over 3 days to develop a core group of facilitators for the study. The first 2 days involved understanding the program's methodology, learning more about the activities and the opportunity to practice activities for feedback from their colleagues; the final day involved a pilot with boys from a school district not involved in the study who provided feedback on the curriculum for further refinement. Because *Jump Start* is an established program with an experienced facilitator, an initial training for the facilitator of the control group was not necessary.

Subsequent trainings for both the control and experimental programs are primarily comprised of one on one and/or small group mentorships, using an apprenticeship model, where the facilitator(s) shadow and work alongside an experienced lead facilitator at a site, for the entire 18-hour curriculum. This model enables facilitators to pair with a lead facilitator and work their way from observing a discussion and/or session to co-facilitating to finally leading a complete session on their own, through an iterative process encompassing a series of check-ins, evaluations, and feedback. During the observation process, facilitators are encouraged to experience and examine all elements of the program, particularly activity and session flow and timing and the dynamic between facilitators and participants and amongst participants themselves. In addition, during the observation period, facilitators are required to review and become familiar with the curriculum. Depending on experience and comfort level, facilitators are welcomed to engage in discussion. Facilitators are deemed ready to lead a session if they participate as a co-facilitator, are observed leading each activity, and if the feedback forms from youth and fidelity checks conducted by research assistants consistently show adherence to program content. This approach to training community-based facilitators creates a longer-term sustainability plan, with lead facilitators training newly interested facilitators from their community.

After each session, the facilitator reconvenes with the lead facilitator to debrief and examine implementation. Participant engagement and behavior, program delivery and timing, and learning objectives are priority topics addressed during debriefing. These elements are also captured on fidelity forms completed by research assistants during each program session. Post-session development also includes biweekly check-ins with project coordinators to analyze feedback and fidelity forms. These meetings ensure maintenance of fidelity and program goals, in a timely fashion. If facilitators need to improve on the delivery of a certain activity or topic or if there were issues with engagement, immediate action is taken to directly address the situation and work towards better implementation for the subsequent session.

Separate conferences (generally every two to three months) are held for job skills intervention facilitators to support facilitators in their development. All facilitators are required to attend these conferences to share their perspectives on the program and also receive collective feedback on overall implementation. Conferences allow for team-building and training. Lastly, extra source materials such as videos, websites, articles, and other media are consistently provided or recommended to support development of facilitator knowledge and skills and keep content up to date.

Process Evaluation Data Collection

Data are collected to assess the quality of program implementation. Research assistants are present at each intervention session and complete a fidelity form to ensure consistent implementation of the intervention or control program as intended as well as unforeseen barriers to implementation. These feedback forms are reviewed by the lead facilitators and PI regularly to provide immediate feedback to facilitators should mid-point corrections be needed.

Youth also complete a feedback form at the end of each session which is reviewed by the facilitators and research assistant to gauge youth interest and engagement in the topics and to make mid-point adjustments to program content and delivery as needed.

In addition to the end of program survey that encourages youth to provide feedback on the entire 18-hour curriculum, after completion of the T3 (final) follow up survey, youth in the intervention arm are invited to participate in a semi-structured interview about their experiences with the program. Interviews with site leads and facilitators provide additional feedback on the program to guide ongoing implementation including sustainability in the participating neighborhoods. Collectively, process evaluation data will be used to inform and improve the intervention content and implementation guidance.

6.3 MEASURES TO MINIMIZE BIAS: RANDOMIZATION AND BLINDING

Assignment of Interventions
Randomization

Randomization was performed at the neighborhood level (i.e., cluster) to reduce risk for contamination. The initial randomization included 10 clusters that were assigned to experimental or control conditions. The study statistician performed this randomization, stratifying by lead site in that neighborhood (YMCA, Urban League, or Other), such that within each stratum, each site/neighborhood had a 50/50 chance of being assigned to intervention or control. Due to a combination of lower-than-expected recruitment by neighborhood (target=96 participants each) and the interest from other community partners, we individually randomized an additional 11 neighborhoods, stratified by type of site. This resulted in a total of 21 clusters randomized, with 11 assigned to experimental and 10 assigned to control. All neighborhoods in the study met the original criteria of being socially or economically disadvantaged and/or predominantly African American.

Blinding

Randomization was performed after approval for the study was obtained for a site in a new neighborhood so that the randomization assignment would not influence a site's willingness to participate. Notably, the PI was blinded from randomization until she had successfully recruited a site to participate in the study. Due to the study design, investigators, research staff, community partners, facilitators, and youth participants could not be blinded to study assignment.

6.4 STUDY INTERVENTION/EXPERIMENTAL MANIPULATION ADHERENCE

Retention in Program

Once community partners recruit youth, retention throughout the 18-hour curriculum is a key focus. Upon enrolling in either the control or experimental arm, prospective participants received program information, signed an assent form and completed a contact information sheet. Research assistants ensure that the document is legible and that all fields are completed. Research assistants use this information to contact the participant prior to each session to remind them of the session. If youth are not present at the beginning of a session, research assistants and facilitators will contact the participant to encourage them to join late. Retention is facilitated by collecting detailed contact information and offering incentives for survey completion ($50 total for baseline, feedback surveys throughout the program, and EOP; $30 for T2; $50 for T3) (see Figure 1 for study flow).

6.5 CONCOMITANT THERAPY

N/A

6.5.1 RESCUE THERAPY

N/A

7 STUDY INTERVENTION/EXPERIMENTAL MANIPULATION DISCONTINUATION AND PARTICIPANT DISCONTINUATION/WITHDRAWAL

7.1 DISCONTINUATION OF STUDY INTERVENTION/EXPERIMENTAL MANIPULATION

A participant may stop participating and withdraw from the study at any time. If a subject discontinues from either arm but not from the study, remaining study procedures will be completed as indicated by the study protocol.

Additionally, a participant may be considered too disruptive for participation in the program (control or intervention) and be asked to discontinue their attendance in the program.

The data to be collected at the time of study intervention discontinuation may include the reason(s) for discontinuing from the intervention, and methods for determining the need to discontinue

7.2 PARTICIPANT DISCONTINUATION/WITHDRAWAL FROM THE STUDY

Participants are free to withdraw from participation in the study at any time upon request. An investigator may discontinue a participant from the study for the following reasons:

- Request from parent or adult caregiver to discontinue their participation
- Lost-to-follow up; unable to contact subject (see **Section 7.3, Lost to Follow-Up**)
- Any event or medical condition or situation occurs such that continued collection of follow-up study data would not be in the best interest of the participant
- The participant meets an exclusion criterion (either newly developed or not previously recognized) that precludes further study participation, i.e., moving out of Pittsburgh

The reason for participant discontinuation or withdrawal from the study will be recorded. Any subject that withdraws from the study, before any data collection is completed, such as survey responses, consent and assent forms will be kept and filed with withdrawn/declined records.

7.3 LOST TO FOLLOW-UP

A participant will be considered lost to follow-up if he fails to respond to at least 10 contact attempts for follow up surveys and the 3 months window for follow up survey completion has passed.

Missing sessions (not participating in the program) will not be considered a reason for loss to follow up. We will make every attempt to reach participants who are missing sessions including using all contact information provided as well as through the community facilitators in their neighborhood.

Before a participant is deemed lost to follow-up, the investigator or research team member will make every effort to regain contact with the participant. These contact attempts will be documented in the participant's study file. Should the participant continue to be unreachable, he will be considered to have withdrawn from the study with a primary reason of lost to follow-up.

8 STUDY ASSESSMENTS AND PROCEDURES.

8.1 ENDPOINT AND OTHER NON-SAFETY ASSESSMENTS

Assent and Consent
Adolescent males ages 13-19 receive a description about the research study and parental letter about the study from the community sites. The parent letter include an option for parents/caregiver to decline their child's participation. We received a waiver of parental permission and waiver of signed consent from the University of Pittsburgh Human Subjects Research Protection Office. Research assistants review the verbal consent form with youth at the beginning of the first session and answer any questions pertaining to confidentiality, the program flow, and survey time points. The consent form covers all 3 waves of data collection, Time 1 through Time 3 as described earlier.

Data Collection
There are several points of data collection throughout the study, starting at baseline all the way through T3: baseline surveys, feedback forms, End of Program (EOP) survey, T2 and T3 follow up surveys. The surveys are all anonymous, linked by a personal study code that youth create by answering a series of questions that only they know the answer to at the beginning of each survey. This method of using a personal study code was selected to ensure anonymity and increase the likelihood of honest responses.

In addition to survey data, other sources of data for this study (primarily for process evaluation and assessing intervention fidelity) include: 1) feedback forms completed by youth after the end of each session; 2) fidelity forms completed by research assistants at each session; 3) interviews with site leads and facilitators; 4) confidential interviews with youth (after Time 3 data collection).

Main Study Phase:
Baseline survey: Youth are asked to complete the baseline survey the first time they attend a session (can enter study at Sessions 1 – 3; if participant is interested in joining, they are asked to return for the next round). All sites conduct web-based surveys (back up paper surveys are used as needed) on tablets using REDCap, an online data management and survey system. Responses to the anonymous web-based secure survey are entered by the youth participants themselves on an electronic tablet; no data are stored on the computers themselves. Only research staff who have been added to the project can access this online database. Data are downloaded and stored on a password-protected share drive that can only be accessed by users with the appropriate permissions. No names are connected to the survey data as each participant creates their own secret code as described above.

End of program survey: At the end of the program, youth are asked to complete an end of program survey that asks about which sessions they attended, their impressions of the program, as well as some additional school and community related questions (about connectedness to school, suspensions, social supports, important adults). Surveys are self- administered on electronic tablets provided by the research staff (back up paper surveys will be used if tablets are not available or fail). All participants, regardless of amount of curriculum completed, are eligible to take this survey; participants are asked to complete the end of program survey later if they miss the last session.

Follow up surveys (T2 and T3):
Participation in the 3- and 9-month follow-up surveys (T2 and T3 surveys, respectively) are facilitated via tracking of participants with the help of community partners at each site. Youth provide detailed contact information at baseline to facilitate follow up. Contact information is confirmed again at sessions following the baseline survey, and at the T2 survey. Youth are also called or texted periodically by research assistants between follow-up surveys to ensure that contact information is still valid. Finally, for those that miss an EOP or T2 survey in the appropriate time frame, a comprehensive “make-up” survey is offered (with the same monetary compensation) to update contact information and increase the likelihood they’ll participate in the next survey.

Follow up surveys are administered approximately three months (T2) and nine months (T3) after the end of the program. Surveys are offered in English and are completed by youth on electronic tablets provided by the research staff (back up paper surveys will be used if tablets are not available or fail). T2 and T3 surveys are similar to the survey administered in Time 1 and aim to assess intervention effects on knowledge, attitudes, self-efficacy, and behaviors regarding addressing disrespectful behaviors toward women and girls among adolescent males. Community sites where the program was held will facilitate reconvening participating adolescents and have agreed to convene these groups of adolescents (regardless of whether they have continued involvement with that site). All participants, regardless of amount of curriculum completed, are eligible to take these surveys.

Emailed/text survey option: Participants who cannot attend the study site or other community site (e.g., due to lack of transportation, neighborhood barriers) for EOP or follow up survey administration will be offered the option to take the survey via email or a texted link on an internet-capable device (e.g., computer, smartphone). The RA will confirm via phone that the participant is OK to take the survey. The email address from our study team will say EHM@chp.edu (or an RA work phone number if the link is texted to participant) and RA number to call or text with any questions or concerns. The link will be specific to the email address that the young person provides and will only allow the survey to be taken

once. Using REDCap's anonymous survey feature, we can create an individualized link, send to participant's email or phone, and see if their survey was completed, but we cannot determine which survey data corresponds to that participant (i.e., remains anonymous). Phone call option: participants can also opt to complete the survey during a phone call with a research assistant, who reads aloud the questions and the participant responds; the research assistant enters the answers directly into the online survey tool.

Alternate contact information: We will also use the alternate contact information provided by youth upon study enrollment to reach them for follow up surveys. This may include, but is not limited to, phone numbers or email addresses for parents, caregivers, other family members, and close friends. We then ask these contacts to facilitate us reaching the participant. Community site partners from the program are also contacted to ask for retention assistance.

Interviews: At or after Time 3, some youth will be invited to participate in a confidential interview about their experiences receiving the control or intervention, their feedback about the relevance of the program, and additional input about the impact of the program on themselves and their peers. The interviews will be digitally recorded, transcribed, and the audiofiles destroyed once the transcription has been checked for accuracy and all identifying information removed.

Follow up procedure for youth who are in placement/incarcerated: Research staff may contact the youth's parents/caregivers through the contact information the youth have provided to arrange administration of follow up surveys per protocol. When the research staff learns from the parent/caregiver, another individual or agency on the contact information sheet, or from the site lead where the program was originally held that a participant is in placement (i.e., at a detention center or locked facility), the research team will offer several options for conducting the follow up survey. 1) The adult caregiver/parent who has contact with the participant can provide information to the participant about how to complete the follow up survey (the research team will provide their contact information); 2) Dr. Miller can send a message to the placement site to convey a message to the participant ([Name of participant] is due to complete a survey as part of a young men's study. This is completely voluntary. Please provide this message to the participant. For example, "Dr. Miller is trying to reach you to ask you to complete the follow up survey from the young men's program. You can reach her at 412-543-8789 or by emailing her at *elizabeth.miller@chp.edu*."); 3) If the detention center/placement facility is willing, and the participant is interested in completing the survey while incarcerated, the research team will come to the facility to meet the participant at a time designated by staff. The survey will be administered on a password protected, wireless computer notepad managed by the research team in a private area designated by staff at the facility; 4) the participant is interested in completing the survey while incarcerated, and the staff at the site are willing to assist, the staff will receive a link to the survey and the young person will complete the survey online on a designated computer. Staff will clear the browsing history on the computer. The youth will receive their incentive after they have been released from placement.

Data Management

Baseline and follow-up survey participation coincide with the beginning of the intervention (Time 1), end of the program (EOP), and three (T2) and nine (T3) months following the end of the 18 hour program (i.e., round). All sites conduct web-based surveys (back up paper surveys are used as needed) on tablets using REDCap, an online data management and survey system. Participation in the 3- and 9-month follow-up surveys (T2 and T3 surveys, respectively) are facilitated via tracking of participants with the help of community partners at each site. Youth provide detailed contact information at baseline to facilitate follow up. Contact information is confirmed again at sessions following the baseline survey, and at the T2 survey. Youth are also called or texted periodically by research assistants between follow-up surveys to ensure that contact information is still valid. Finally, for those that miss an EOP or T2 survey in the appropriate time frame, a comprehensive "make-up" survey is offered (with the same monetary

compensation) to update contact information and increase the likelihood they'll participate in the next survey.

Responses to the anonymous web-based secure survey are entered by the youth participants themselves on an electronic tablet; no data are stored on the computers themselves. Only research staff who have been added to the project can access this online database. Data are downloaded and stored on a password-protected share drive that can only be accessed by users with the appropriate permissions. No names are connected to the survey data as each participant creates their own secret code as described above.

The only study documents that contain unique personal identifiers are contact forms and the contact list of participants (youth and prevention educators) that are kept to assist with re-contacting participants for follow up surveys). Contact forms are stored in a secure file drawer inside the locked office of the PI whenever not in use. Contact forms are stored separately from any survey data collected in this study (the survey data are collected via computer and immediately housed in a password-protected secure database). The names of participants are kept in encrypted files on a password-protected server behind the UPMC firewall.

Outcomes

All outcomes are collected via self-report on anonymous surveys by participants.

Primary Outcome:

SV/ARA perpetration: At baseline and T3 (9 months post-intervention), participants report whether they perpetrated the following SV or ARA behaviors in the last 9 months. The primary outcome measure for the study is <u>any</u> report of SV or ARA perpetration, i.e., yes to any of the following items.

The following two constructs focus on ARA behaviors against a dating partner. "Any ARA perpetration" is measured as a yes to any of these 13 items.

Physical/Sexual relationship violence: Three items are used to assess physical or sexual violence perpetration against a partner or ex-partner.(33) Participants report whether they performed each action, which is dichotomized as yes to any. Examples include "hit, pushed, slapped, choked or otherwise physically hurt someone you were going out with or hooking up with?" and "used physical force or threats to make someone you were going out with or hooking up with have sex (vaginal, oral, or anal sex) when they didn't want to?"

Dating abuse: A ten-item scale, developed for use with high school-aged students, is used to assess whether the participant perpetrated any abuse against a dating partner (34). Examples include "convinced them to have sex, after they said no a few times" and "told them not to talk to others or told them who they could hang out with." A positive response to any of these items is counted as any ARA perpetration.

"Any SV perpetration" is measured as yes to any of the sexual relationship violence and dating abuse items above as well as yes to any of the behaviors below.

Non-partner sexual violence: To measure whether a participant committed sexual violence against a *non-partner*, the two sexual IPV items were modified to query for people they had NOT gone out with or hooked up with, and included friends, family, and strangers (33). Responses are dichotomized as yes to any.

Incapacitated sex: Participants are asked if they had done something sexual with someone when that person was "too drunk or high to stop you,"(35) with a response of "yes" coded as yes to incapacitated sex.

Use of drugs or alcohol on purpose for sex: Participants are asked whether they had purposely given someone alcohol or drugs to do something sexual with that person(36). A response of "yes" is coded as yes to use of drugs or alcohol on purpose for sex.

Sexual harassment: Five items assess the frequency with which a participant has engaged in sexual harassment against someone from "making unwanted sexual comments" to "touching or grabbing them in a sexual way."(37,38) Any endorsement of these behaviors is coded as yes for sexual harassment.

Cyber sexual abuse: Given the ubiquity of social media and smartphones, three items assess for frequency of sexual harassment, including "try to get them to talk about sex when they did not want to"

and "post or publicly share a nude or semi-nude picture of them" using mobile apps, social networks, texts, or other digital communication.(3,39,40) Any endorsement is coded as yes for cyber sexual abuse.

Secondary Outcomes:

Positive bystander intervention behaviors: A scale developed for use with high school students is used to determine whether participants will intervene or interrupt in a positive manner when they witness disrespectful or abusive behaviors by peers(34). The scale first assesses whether participants had witnessed nine different abusive behaviors among their peers (e.g., "making rude or disrespectful comments about a girl's body, clothing, or make-up"). For each behavior witnessed, participants are asked if they performed three positive behaviors (e.g., "I talked to an important adult about it"). Reporting at least one positive response per behavior witnessed is summed for the 9 items, creating a maximum summary score equal to 9.

Condom negotiation self-efficacy: A 5-item scale is used to assess how confident participants feel about negotiating condom use with a partner(41). Three positive (e.g., "I feel confident in my ability to suggest using condoms with a new partner") and two negative (e.g., "If I were unsure of my partner's feelings about using condoms, I would not ask my partner to use one"; reverse coded) are used. Responses are on a 5-point Likert scale, with values from "strongly disagree" to "strongly agree." The scale is analyzed using the mean score.

Attitudes related to condom and contraceptive use: A 10-item scale evaluating the participants' temperament towards the usage of condoms and other contraceptive modalities. Examples of these 10 items include,(42–45) "using birth control makes sex feel unnatural" and "I am in favor of my partner and me using birth control. The attitudes are measured by utilizing a 5-point Likert scale from "strongly disagree" to "strongly agree" and a mean score is calculated; a higher score indicates a more positive attitude towards condom and contraceptive use.

Recognition of ARA : Recognition of abusive behaviors is measured with a 12-item scale that addresses the ability of participants to recognize offensive and harmful actions against a partner as abusive (46); for example, "name calling or insulting them" and "threatening to hit them". Responses range on a 5-point Likert scale from "strongly disagree" to "strongly agree." A mean score across the 12 items is calculated, with the higher score indicating higher recognition of abusive behaviors.

Gender equitable attitudes : A 13-item scale is utilized to measure participants' views on gender- equitable norms (34,47,48) , such as, "A guy never needs to hit another guy to get respect" and "I would be friends with a guy who is gay". Response options are on a 5-point Likert scale from "strongly disagree" to "strongly agree" and a mean score across the 13 items is calculated. A higher mean is indicative of more equitable attitudes.

Intentions to intervene with peers: Utilizing eight items, this attitudinal measure assesses the likelihood for a participant to intervene when witnessing a range of harmful behaviors amongst male peer students, similar to the scenarios for assessing actual bystander intervention behaviors described above (34). Responses are on a 5-point Likert scale from "very unlikely" to "very likely" and a mean score across the eight items is calculated; a higher score indicates greater intentions to intervene.

8.2 SAFETY ASSESSMENTS

Data Security: Responses to the anonymous web-based secure survey are entered by the youth participants themselves through a computer-based system; the data are automatically entered into a password protected data base accessible only to the investigative team. No names are connected to the survey data as each participant creates their own secret code as described above. The only study documents that will contain unique personal identifiers are consent forms and the contact list of participants that are kept to assist with re-contacting participants at the end of the intervention (Time 2) and in the subsequent year (Time 3). Contact forms will be stored in a secure file drawer inside the locked office of the PI's research lab whenever not in use. Contact forms will be stored separately from any

survey data collected in this study (the survey data are collected via computer and immediately housed in a password-protected secure database).The names of participants will be kept in an encrypted file on a password protected secure on-line server (available to the research team through the University of Pittsburgh), and accessed only when needed to arrange the follow up data collection with each community site(Time 2 and Time 3). Please note that there are three layers of protection for the contact information: password protection to enter the University of Pittsburgh system, a username that has been granted access to the secure drive, and another password to decode the encrypted file. This information will be accessed only when needed to arrange follow up contact with participants and scheduling data collection.

Internal Data and Safety Monitoring Plan. During the course of conducting other research studies regarding violence prevention programs, specifically a randomized control trial with over 2000 youth athletes (using similar survey measures), we did not experience any adverse events, including no evidence of emotional distress among participants and no disclosures of abuse or violence. However, given the sensitivity of the questions being asked regarding violence perpetration, we are taking extra precautionary measures with an internal data safety and monitoring plan in place. The senior research coordinator is responsible for daily monitoring of data and safety. They will work with research assistants, to ensure that all data are collected and stored securely. The research team consisting of the PI (Miller), research coordinator, research assistants, data analysts, and other research staff as appropriate, will meet weekly (as possible) to review study progress and status of data collection and safety of participants. Each neighborhood/site has a designated point RA, who along with the research coordinator, is connected to the community site leads and can identify concerns about data collection or participant safety.

Given the sensitivity of the questions being asked regarding violence perpetration, we received a Certificate of Confidentiality from the Centers for Disease Control and Prevention to protect the research data from subpoena.

Extra precautionary measures were taken to protect the data, including the use of a personally created ID code to maintain anonymity of the survey data and an internal data safety and monitoring plan, which included the following:

a) Systematically review assessment materials to ensure that assessment is conducted appropriately and that participants disclosing abuse or violence during the course of taking the survey receive appropriate connection to violence-related services and that mandated reports are made by site personnel when appropriate.

b) Systematically review notes from research assistants to ensure that participants experiencing distress are being connected directly with the site directors and youth workers, receiving educational materials, and being referred appropriately; this includes ensuring that all research assistants document asking each participant about emotional distress after completion of the survey.

c) Monitor staff performance with regard to protection of privacy, confidentiality, maintenance of secure data bases, and study procedures designed to reduce the risk of distress and potential breaches of confidentiality.

d) Ensure that the PI (Miller), or a designated qualified individual, will be available by pager in case research staff needs to confer regarding participants' behaviors or comments made during a survey or other research activities.

e) Ensure that the PI (Miller), or a designated qualified individual, will be available by pager in case educators or violence prevention advocates from Center for Victims and Pittsburgh Action Against Rape, needs to confer regarding participants' or youth workers' behaviors or comments made during study implementation (i.e., during training, survey administration, or follow up contact with site administrators, youth workers and facilitators).

f) Review and report any adverse events associated with the study.

8.3 ADVERSE EVENTS AND SERIOUS ADVERSE EVENTS

8.3.1 DEFINITION OF ADVERSE EVENTS

This study is of minimal risk to participants because there is low likelihood of any risk or adverse events to occur during administration of anonymous surveys and confidential interviews. Precautions will be implemented to protect participating subjects' privacy and confidentiality. The primary risks are risk for breach of confidentiality and potential for emotional distress related to answering survey questions, both highly unlikely events.

Should a participant disclose safety concerns during the course of programming, the site RA follows the emotional distress protocol and immediately notifies Dr. Miller (PI), a plan for safety and any reporting requirements are addressed, and the RA completes a report. Such events are reported to the IRB.

The senior research coordinator is responsible for daily monitoring of data and safety. They will work with research assistants s, to ensure that all data are collected and stored securely. The research team consisting of the PI (Miller), research coordinator, research assistants, data analysts, and other research staff as appropriate, will meet weekly (as possible) to review study progress and status of data collection and safety of participants.

8.3.2 DEFINITION OF SERIOUS ADVERSE EVENTS

We follow the definition of serious adverse events as outlined by the University of Pittsburgh Human Subjects Research Protection Office, and any questions we have about specific events, we review with a representative from this office.

While we do not anticipate any serious adverse events, we will promptly report to the IRB any unintended or unanticipated consequences from participating in this study. If any serious adverse event occurs (death, life threatening, new, serious, or permanent disability), it will be reported within 72 hours to the IRB. Specific information that will be recorded on the adverse event form will include details of the adverse event, treatment required for the event, the participant's condition after the event, an estimate of the extent of injury, and ways to prevent similar events from occurring in the future. Dr. Miller will classify the relationship of the study protocol to the event on a scale from not related to highly probably related as outlined by the IRB, and severity of the event from mild to severe based on a degree of intensity outlined by the IRB, which will be reviewed by the IRB.

All adverse events will be reported to Centers for Disease Control and Prevention in addition to the University of Pittsburgh Human Research Protection Office.

8.3.3 CLASSIFICATION OF AN ADVERSE EVENT

8.3.3.1 SEVERITY OF EVENT

The following guidelines will be used to describe severity.

- **Mild** – Events require minimal or no treatment and do not interfere with the participant's daily activities.
- **Moderate** – Events result in a low level of inconvenience or concern with the therapeutic measures. Moderate events may cause some interference with functioning.
- **Severe** – Events interrupt a participant's usual daily activity and may require systemic drug therapy or other treatment. Severe events are usually potentially life-threatening or incapacitating. Of note, the term "severe" does not necessarily equate to "serious".

8.3.3.2 RELATIONSHIP TO STUDY INTERVENTION/EXPERIMENTAL MANIPULATION

All adverse events (AEs) will have their relationship to study procedures, including the intervention, assessed by an appropriately-trained clinician (Dr. Miller, PI) based on temporal relationship and his/her clinical judgment. The degree of certainty about causality will be graded using the categories below.

- **Related** – The AE is known to occur with the study procedures, there is a reasonable possibility that the study procedures caused the AE, or there is a temporal relationship between the study procedures and the event. Reasonable possibility means that there is evidence to suggest a causal relationship between the study procedures and the AE.
- **Not Related** – There is not a reasonable possibility that the study procedures caused the event, there is no temporal relationship between the study procedures and event onset, or an alternate etiology has been established.

8.3.3.3 EXPECTEDNESS

Given the sensitivity of the questions being asked regarding violence perpetration, we are taking extra precautionary measures with an internal data safety and monitoring plan in place, described above. All research personnel are trained on the emotional distress protocol and complete training on child abuse reporting requirements. Each neighborhood/site has a designated point RA, who along with the research coordinator, is connected to the community site leads and can identify concerns about data collection or participant safety.

A Summary Report of the Data and Safety Monitoring Plan will be submitted with the annual IRB renewal.

8.3.4 TIME PERIOD AND FREQUENCY FOR EVENT ASSESSMENT AND FOLLOW-UP

The occurrence of an adverse event (AE) or serious adverse event (SAE) may come to the attention of study personnel during study visits and interviews of a study participant.

Should a participant disclose safety concerns during the course of programming or study procedures, the site RA follows the emotional distress protocol and immediately notifies Dr. Miller (PI), a plan for safety and any reporting requirements are addressed, and the RA completes a report. Such events are reported to the IRB.

All AEs occurring while on study will be documented appropriately regardless of relationship. All AEs will be followed to adequate resolution.

8.3.5 ADVERSE EVENT REPORTING

Review and report any adverse events associated with the study to the IRB as well as to the Centers for Disease Control and Prevention.

8.3.6 SERIOUS ADVERSE EVENT REPORTING

In consultation with the PI, a trained member of the study team will be responsible for conducting an evaluation of a serious adverse event and shall report the results of such evaluation to the CDC and the reviewing Institutional Review Board (IRB) as soon as possible, but in no event later than 10 working days after the investigator first learns of the event.

8.3.7 REPORTING EVENTS TO PARTICIPANTS

N/A

8.3.8 EVENTS OF SPECIAL INTEREST

N/A

8.3.9 REPORTING OF PREGNANCY

N/A

8.4 UNANTICIPATED PROBLEMS

8.4.1 DEFINITION OF UNANTICIPATED PROBLEMS

This protocol uses the definition of Unanticipated Problems as defined by the Office for Human Research Protections (OHRP). OHRP considers unanticipated problems involving risks to participants or others to include, in general, any incident, experience, or outcome that meets **all** of the following criteria:

- Unexpected in terms of nature, severity, or frequency given (a) the research procedures that are described in the protocol-related documents, such as the Institutional Review Board (IRB)-approved research protocol and informed consent document; and (b) the characteristics of the participant population being studied;
- Related or possibly related to participation in the research ("possibly related" means there is a reasonable possibility that the incident, experience, or outcome may have been caused by the procedures involved in the research); and
- Suggests that the research places participants or others at a greater risk of harm (including physical, psychological, economic, or social harm) than was previously known or recognized.

8.4.2 UNANTICIPATED PROBLEMS REPORTING

The investigator will report unanticipated problems (UPs) to the reviewing Institutional Review Board (IRB). The UP report will include the following information:

- Protocol identifying information: protocol title and number, PI's name, and the IRB project number
- A detailed description of the event, incident, experience, or outcome
- An explanation of the basis for determining that the event, incident, experience, or outcome represents an UP
- A description of any changes to the protocol or other corrective actions that have been taken or are proposed in response to the UP

To satisfy the requirement for prompt reporting, UPs will be reported using the following timeline:

- UPs that are serious adverse events (SAEs) will be reported to the IRB within 24 hours of the investigator becoming aware of the event
- Any other UP will be reported to the IRB within 10 days of the investigator becoming aware of the problem
- All UPs will be reported to appropriate institutional officials (as required by an institution's written reporting procedures), the supporting agency head (or designee), and the Office for Human Research Protections (OHRP) in accordance with policy of the IRB's receipt of the report of the problem from the investigator.

8.4.3 REPORTING UNANTICIPATED PROBLEMS TO PARTICIPANTS

Include content in this section if applicable, otherwise note as "N/A." Describe how participants will be informed about UPs on an individual or aggregate level.

N/A

9 STATISTICAL CONSIDERATIONS

9.1 STATISTICAL HYPOTHESES

Aim 1: To test, via a 2-arm cluster-randomized trial in youth-serving agencies, the effectiveness of "Manhood 2.0," an 18 hour SV/ARA prevention program compared to an 18 hour life skills development curriculum. Approximately 3 months after end of program implementation), compared to controls, youth will demonstrate increased positive bystander intervention behaviors (Secondary outcome, H1.1). Intermediate outcomes are: increased condom and contraceptive self-efficacy (H1.2); increased recognition of abusive behaviors (H1.3); increased gender-equitable attitudes (H1.4); and increased intentions to intervene with peers (H1.5).
Aim 2: To examine whether "Manhood 2.0" results in sustained improvements in these same Aim 1 outcomes assessed at approximately 9 months after end of program implementation (Time 3), as well as reductions in self-reported perpetration of SV and ARA (Primary Outcome) toward females from baseline to Time 3, compared to controls.

Aim 3: To explore whether demographics, participants' pre-intervention risk and protective factors (e.g., history of SV/ARA exposure, sexual risk, connectedness), and setting-level characteristics (e.g., staff experience, organizational capacity) are associated with differences in outcomes.

9.2 SAMPLE SIZE DETERMINATION

Power and sample size calculations were based on clinically meaningful differences between treatment groups with respect to changes in the outcomes across time (i.e. intervention effect). For the primary outcome - any SV/ARA perpetration at Time 3, the detectable difference between arms was calculated based on traditional methods that assumed a fixed number of clusters as well as fixed number of subjects per cluster.(49) Twenty-one clusters were randomized, assuming a within-cluster intra-class correlation (ICC) of 0.01 (within-school correlations for abuse perpetration, similar to our team's prior work with a related sexual violence prevention program with male athletes(34,50)), and a 20% baseline SV perpetration rate in the control arm. With 866 participants (approximately 41 boys at each site) and an 80% retention rate at Time 3, we expect to have 80% power to detect an 8.3 point decrease in SV perpetration (42% relative decrease) due to the intervention. We anticipate having ample power to detect clinically meaningful changes in bystander behaviors and the secondary outcomes as well. Based on previous studies, the within-cluster ICCs for each of our secondary outcomes ranged from 0.006 to 0.01. If we assume the upper end of that range, we will have at least 80% power to detect standardized mean differences as small as 0.23 between study arms.

9.3 POPULATIONS FOR ANALYSES

Intention to Treat (ITT) Analysis Population: All enrolled participants
Per-protocol Analysis (see exploratory analyses): All participants who received sufficient 'dose' of the program

9.4 STATISTICAL ANALYSES

9.4.1 GENERAL APPROACH

Generalized linear mixed models will be used to account for the correlation among youth from the same cluster as well as the correlation between observations from the same youth. Descriptive statistics will be used to summarize the sample with regard to baseline characteristics of interest. Means and standard deviations will be presented for continuous variables, while sample proportions will be provided for categorical variables. 95% confidence intervals will accompany all sample statistics. Primary assessment of intervention effects will be based on intent-to-treat estimates. As-treated, or treatment-on-the-treated (TOT), effect parameters will be estimated in secondary analyses and reported as exploratory. Between-site differences regarding intervention effects will be assessed based on level of staff/facilitator engagement in curricular delivery as well as other observed external factors that may interact with the intervention to alter outcomes. SAS software will be used for all statistical analyses.

To assess differences at baseline between the youth in the experimental and control groups, demographics such as grade-level, race, nativity, and parental education will be compared while accounting for within-neighborhood clustering. Demographic variables as well as neighborhood-level characteristics resulting in between-arm imbalances will be considered as covariates in the primary and secondary analyses.

Participation bias will be assessed by comparison of age and race/ethnicity of youth participating in the study compared to the overall demographics of adolescents (school district and census data). Significant differences detected via chi-square analysis will be noted as potential validity threats.

An attrition analysis will be conducted by comparing youth who completed follow-up surveys with those who did not with regard to demographics as well as outcomes measured at baseline. All hypotheses will be two-sided tests with a significance level of 5%.

Missing data were minimized as much as possible by keeping the surveys as short as possible to reduce survey burden, encouraging youth to be as complete and honest as possible by ensuring anonymity of the surveys, and working assiduously with community partners to ensure that youth stay engaged and can be tracked to complete follow up surveys. Mechanisms for missing data will be investigated by comparing important covariates between youth with and without missing data at each time point. We will characterize these mechanisms as 1) missing completely at random (MCAR), 2) missing at random (MAR), or not missing at random (NMAR). If the nature of our missing data is ignorable (either MCAR or MAR), we will utilize imputation methods such as multiple imputation via chained equations (MICE) to handle dropout. Ultimately, sensitivity analyses will be conducted comparing the results of our imputation methods to complete-case and available-data analyses.

9.4.2 ANALYSIS OF THE PRIMARY ENDPOINT(S)

The primary outcome for this study is reductions in self-reported perpetration of SV and ARA at Time 3, compared to controls. We will examine whether Manhood 2.0 results in improvements in the primary outcome assessed at 9 months after end of program (Time 3) compared to controls. Generalized linear mixed models will be used and will include variables for baseline SV/ARA perpetration, treatment group, and random effects for cluster.

9.4.3 ANALYSIS OF THE SECONDARY ENDPOINT(S)

For positive bystander intervention (secondary outcome) and the remaining additional outcomes, the models will include variables for the secondary outcome at baseline, treatment group, and random effects for cluster. This will allow us to assess the effect of the study arm after accounting for clustering within neighborhoods. As an exploratory analysis, all three time points will be included in a single generalized linear mixed model to quantify the long-term trajectories of each of the secondary outcomes.

Following Twisk and Proper's recommendations for randomized controlled trials with both baseline and follow up measures (in this case abuse perpetration),(51) we will also construct multinomial logistic regression models that account for presence or absence of baseline perpetration. That is, we will examine intervention effects among youth reporting baseline SV/ARA perpetration and the likelihood of becoming 'non abusive' at follow up (i.e., an 'early intervention' effect). Similarly, we will examine intervention effects among youth with no baseline SV/ARA perpetration and the likelihood of 'staying non abusive' (i.e., a primary prevention effect). We have used this approach to analyze intervention effects on abuse exposure in our school health center relationship abuse prevention study.(52)

9.4.4 SAFETY ANALYSES

N/A

9.4.5 BASELINE DESCRIPTIVE STATISTICS

To assess differences at baseline between the youth in the experimental and control groups, demographics such as grade-level, race, nativity, and parental education will be compared while accounting for within-neighborhood clustering. Demographic variables as well as neighborhood-level characteristics resulting in between-arm imbalances will be considered as covariates in the primary and secondary analyses.

9.4.6 PLANNED INTERIM ANALYSES

N/A

9.4.7 SUB-GROUP ANALYSES

Participation bias will be assessed by comparison of age and race/ethnicity of youth participating in the study compared to the overall demographics of adolescents (school district and census data). Significant differences detected via chi-square analysis will be noted as potential validity threats.
An attrition analysis will be conducted by comparing youth who completed follow-up surveys with those who did not with regard to demographics as well as outcomes measured at baseline. All hypotheses will be two-sided tests with a significance level of 5%.

9.4.8 TABULATION OF INDIVIDUAL PARTICIPANT DATA

N/A

9.4.9 EXPLORATORY ANALYSES

We will also conduct two sets of exploratory analyses. First, we will conduct an intensity-adjusted analysis that reflects the actual delivery of the program. To achieve this, we will use a continuous score of "intensity" to replace the binary intervention variable in the models for the primary analysis. This score will be calculated for each round (program delivery) by using two sources of information. The first is information collected systematically about each session by trained research assistants who observed the sessions, including whether each task assigned to a module was performed (yes or no). The second is a summary measure of overall attendance for each round, tracked by using sign-in sheets with the research assistants. The second set will be a per-protocol analysis. This set of models will include only participants in the intervention arm that received the full intervention, defined as having covered a minimum threshold of tasks across the six sessions and having sufficient attendance. These measures replicate the intensity score values, but are then dichotomized to yes or no to receiving the full intervention. All control arm participants will be included in these models.

To explore whether demographics, youth pre-intervention risk and protective factors (e.g., history of SV/ARA exposure, sexual risk, connectedness), and site-level differences (e.g., staff experience, organizational capacity, intervention intensity) moderate the effect of the intervention on the primary and secondary outcomes, linear mixed models slightly different from those described above will be utilized. The outcome variables will be modeled as a function of the following variables: the outcome at baseline, the treatment group, the potential moderator, the interaction between the treatment group and moderator, and a random effect for cluster. A significant interaction suggests the presence of intervention effect heterogeneity, and we will follow the approach of Kraemer (53) by focusing on effect size derivation rather than formal hypothesis testing.

10 SUPPORTING DOCUMENTATION AND OPERATIONAL CONSIDERATIONS

10.1 REGULATORY, ETHICAL, AND STUDY OVERSIGHT CONSIDERATIONS

10.1.1 INFORMED CONSENT PROCESS

10.1.1.1 CONSENT/ASSENT AND OTHER INFORMATIONAL DOCUMENTS PROVIDED TO PARTICIPANTS

Assent and Consent
Adolescent males ages 13-19 receive a description about the research study and parental letter about the study from the community sites. The parent letter includes an option for parents/caregiver to decline their child's participation. We received a waiver of parental permission and waiver of signed consent from the University of Pittsburgh Human Subjects Research Protection Office. Research assistants review the verbal assent/consent form with youth at the beginning of the first session and answer any questions pertaining to confidentiality, the program flow, and survey time points. The consent information sheet covers all 3 waves of data collection, Time 1 through Time 3 as described earlier.

10.1.1.2 CONSENT PROCEDURES AND DOCUMENTATION

A waiver to document informed consent and a waiver for parental permission were granted by the IRB.

10.1.2 STUDY DISCONTINUATION AND CLOSURE

N/A

10.1.3 CONFIDENTIALITY AND PRIVACY

Participant confidentiality and privacy is strictly held in trust by the participating investigators, their staff, the safety and oversight monitor(s), and the sponsor(s) and funding agency. This confidentiality is extended to the data being collected as part of this study. Data that could be used to identify a specific study participant will be held in strict confidence within the research team. No personally-identifiable information from the study will be released to any unauthorized third party without prior written approval of the sponsor/funding agency.

All research activities will be conducted in as private a setting as possible.

The study monitor, other authorized representatives of the sponsor or funding agency, representatives of the Institutional Review Board (IRB), or regulatory agencies, may inspect all documents and records required to be maintained by the investigator.

The study participant's contact information will be securely stored with the research team for internal use during the study. At the end of the study, all records will continue to be kept in a secure location for as

long a period as dictated by the reviewing IRB, Institutional policies, or sponsor/funding agency requirements.

Study participant research data, which is for purposes of statistical analysis and scientific reporting, will be stored in the research team's secure, password protected electronic database. This will not include the participant's contact or identifying information. Rather, individual participants and their research data will be identified by a unique study identification number (using the secret code generated by participants).

Measures Taken to Ensure Confidentiality of Data Shared per the CDC Data Sharing Policies
It is CDC policy that the results and accomplishments of the activities that it funds should be made available to the public. The PI will ensure all mechanisms used to share data will include proper plans and safeguards for the protection of privacy, confidentiality, and security for data dissemination and reuse (e.g., all data will be thoroughly de-identified and will not be traceable to a specific study participant). Plans for archiving and long-term preservation of the data will be implemented, as appropriate.

Certificate of Confidentiality
To further protect the privacy of study participants, the PI received a certificate of confidentiality from the Centers for Disease Control and Prevention. Investigators and others who have access to research records will not disclose identifying information except when the participant consents or in certain instances when federal, state, or local law or regulation requires disclosure. NIH expects investigators to inform research participants of the protections and the limits to protections provided by a Certificate issued by this Policy.
Data Security: Responses to the anonymous web-based secure survey are entered by the youth participants themselves through a computer-based system; the data are automatically entered into a password protected data base accessible only to the investigative team. No names are connected to the survey data as each participant creates their own secret code as described above. The only study documents that will contain unique personal identifiers are consent forms and the contact list of participants that are kept to assist with re-contacting participants at the end of the intervention (Time 2) and in the subsequent year (Time 3).
Contact forms will be stored in a secure file drawer inside the locked office of the PI's research lab whenever not in use. Contact forms will be stored separately from any survey data collected in this study (the survey data are collected via computer and immediately housed in a password-protected secure database).The names of participants will be kept in an encrypted file on a password protected secure on-line server (available to the research team through the University of Pittsburgh), and accessed only when needed to arrange the follow up data collection with each community site(Time 2 and Time 3). Please note that there are three layers of protection for the contact information: password protection to enter the University of Pittsburgh system, a username that has been granted access to the secure drive, and another password to decode the encrypted file. This information will be accessed only when needed to arrange follow up contact with participants and scheduling data collection.

10.1.4 FUTURE USE OF STORED SPECIMENS AND DATA

Data collected for this study will be analyzed and stored at the University of Pittsburgh in Dr. Miller (PI) research office, on a password protected, encrypted, secure drive. After the study is completed, the de-identified, archived data will be made available to interested researchers 12 months after publication of the intervention manuscript.

10.1.5 KEY ROLES AND STUDY GOVERNANCE

This study is a cooperative agreement with the Centers for Disease Control and Prevention (CDC). All study procedures and modifications are developed in collaboration with the CDC program officials who have oversight over this study.

Principal Investigator	Study Statistician
Elizabeth Miller, MD, PhD	Kaleab Abebe, PhD
University of Pittsburgh	University of Pittsburgh
120 Lytton Ave., Suite 302, Pittsburgh PA 15213	200 Meyran Ave., Suite 300, Pittsburgh, PA 15213
412-692-8504	412-246-6931
elizabeth.miller@chp.edu	Kza3@pitt.edu

10.1.6 SAFETY OVERSIGHT

As a minimal risk study, safety oversight is under the direction of the PI and an internal safety monitoring group including the study coordinator and study statistician.

10.1.7 CLINICAL MONITORING

Extra precautionary measures are taken to protect the data, including the use of a personally created ID code to maintain anonymity of the survey data and an internal data safety and monitoring plan, which includes the following:

a) Systematically review assessment materials to ensure that assessment is conducted appropriately and that participants disclosing abuse or violence during the course of taking the survey receive appropriate connection to violence-related services and that mandated reports are made by site personnel when appropriate.

b) Systematically review notes from research assistants to ensure that participants experiencing distress are being connected directly with the site directors and youth workers, receiving educational materials, and being referred appropriately; this includes ensuring that all research assistants document asking each participant about emotional distress after completion of the survey.

c) Monitor staff performance with regard to protection of privacy, confidentiality, maintenance of secure data bases, and study procedures designed to reduce the risk of distress and potential breaches of confidentiality.

d) Ensure that the PI (Miller), or a designated qualified individual, will be available by pager in case research staff needs to confer regarding participants' behaviors or comments made during a survey or other research activities.

e) Ensure that the PI (Miller), or a designated qualified individual, will be available by pager in case educators or violence prevention advocates from Center for Victims and Pittsburgh Action Against Rape, needs to confer regarding participants' or youth workers' behaviors or comments made during study implementation (i.e., during training, survey administration, or follow up contact with site administrators, youth workers and facilitators).

f) Review and report any adverse events associated with the study.

10.1.8 QUALITY ASSURANCE AND QUALITY CONTROL

Quality control (QC) procedures will be implemented as follows:

Informed consent --- Study staff will review the verbal consenting process. This review will evaluate compliance with GCP, accuracy, and completeness. Feedback will be provided to the study team to ensure proper consenting procedures are followed.

Source documents and the electronic data --- Data will be initially captured on source documents (see **Section 10.1.9, Data Handling and Record Keeping**) and will ultimately be entered into the study database. To ensure accuracy, staff will compare a representative sample of source data against the database, targeting key data points in that review.

Intervention Fidelity — Consistent delivery of the study interventions will be monitored throughout the intervention phase of the study. Procedures for ensuring fidelity of intervention delivery are described in **Section 6.2.1, Interventionist Training and Tracking**.

Protocol Deviations – The study team will review protocol deviations on an ongoing basis and will implement corrective actions when the quantity or nature of deviations are deemed to be at a level of concern.

Should independent monitoring become necessary, the PI will provide direct access to all trial related sites, source data/documents, and reports for the purpose of monitoring and auditing by the sponsor/funding agency, and inspection by local and regulatory authorities.

10.1.9 DATA HANDLING AND RECORD KEEPING

10.1.9.1 DATA COLLECTION AND MANAGEMENT RESPONSIBILITIES

The investigator will be responsible for ensuring the accuracy, completeness, legibility, and timeliness of the data reported. All source documents will be completed in a neat, legible manner to ensure accurate interpretation of data.

Hardcopies of the contact and attendance sheets as well as fidelity forms will be provided for use as source document worksheets for recording data. Data recorded in the electronic database derived from source documents will be consistent with the data recorded on the source documents.

The data system includes password protection and internal quality checks, such as automatic range checks, to identify data that appear inconsistent, incomplete, or inaccurate. Study data will be entered directly from the source documents.

The main survey data are entered directly by study participants via an electronic notepad displaying an online survey. Those data are transmitted directly into the secure database.

10.1.9.2 STUDY RECORDS RETENTION

Study records will be maintained for 10 years following guidance from the University of Pittsburgh Human Research Protection Office.

10.1.10 PROTOCOL DEVIATIONS

This protocol defines a protocol deviation as any noncompliance with the clinical trial protocol, International Council on Harmonisation Good Clinical Practice (ICH GCP), or Manual of Procedures (MOP) requirements. The noncompliance may be either on the part of the participant, the investigator, or the study site staff. As a result of deviations, corrective actions will be developed by the site and implemented promptly.

These practices are consistent with ICH GCP:

- Section 4.5 Compliance with Protocol, subsections 4.5.1, 4.5.2, and 4.5.3
- Section 5.1 Quality Assurance and Quality Control, subsection 5.1.1
- Section 5.20 Noncompliance, subsections 5.20.1, and 5.20.2.

It is the responsibility of the PI to use continuous vigilance to identify and report deviations within 2 working days of identification of the protocol deviation. Protocol deviations will be sent to the reviewing Institutional Review Board (IRB) per their policies. The PI is responsible for knowing and adhering to the reviewing IRB requirements. Further details about the handling of protocol deviations will be included in the MOP for all research staff.

10.1.11 PUBLICATION AND DATA SHARING POLICY

This study will be conducted in accordance with the following publication and data sharing policies and regulations:

CDC's Public Access Policy, which ensures that the public has access to the published results of CDC funded research. It requires scientists to submit final peer-reviewed journal manuscripts that arise from CDC funds to the digital archive PubMed Central upon acceptance for publication.

While this is CDC-funded, this study will comply with the NIH Data Sharing Policy and Policy on the Dissemination of NIH-Funded Clinical Trial Information and the Clinical Trials Registration and Results Information Submission rule. As such, this trial is registered at ClinicalTrials.gov, and results information from this trial will be submitted to ClinicalTrials.gov. In addition, every attempt will be made to publish results in peer-reviewed journals. Data from this study may be requested from other researchers 10 years after the completion of the primary endpoint by contacting the PI. Considerations for ensuring confidentiality of these shared data are described in Section 10.1.3.

10.1.12 CONFLICT OF INTEREST POLICY

The independence of this study from any actual or perceived influence is critical. Therefore, any actual conflict of interest of persons who have a role in the design, conduct, analysis, publication, or any aspect of this trial will be disclosed and managed. Furthermore, persons who have a perceived conflict of interest will be required to have such conflicts managed in a way that is appropriate to their participation in the design and conduct of this trial. The study leadership in conjunction with the CDC has established policies

and procedures for all study group members to disclose all conflicts of interest and will establish a mechanism for the management of all reported dualities of interest.

10.2 ADDITIONAL CONSIDERATIONS

N/A

10.3 ABBREVIATIONS AND SPECIAL TERMS

AE	Adverse Event
CDC	Centers for Disease Control and Prevention
CMP	Clinical Monitoring Plan
COC	Certificate of Confidentiality
CONSORT	Consolidated Standards of Reporting Trials
CRF	Case Report Form
DHHS	Department of Health and Human Services
eCRF	Electronic Case Report Forms
FFR	Federal Financial Report
GCP	Good Clinical Practice
GLP	Good Laboratory Practices
ICH	International Council on Harmonisation
ICMJE	International Committee of Medical Journal Editors
IDE	Investigational Device Exemption
IND	Investigational New Drug Application
IRB	Institutional Review Board
ITT	Intention-To-Treat
MOP	Manual of Procedures
NCT	National Clinical Trial
NIH	National Institutes of Health
OHRP	Office for Human Research Protections
PI	Principal Investigator
QA	Quality Assurance
QC	Quality Control
SAE	Serious Adverse Event
SAP	Statistical Analysis Plan
SOA	Schedule of Activities
SOP	Standard Operating Procedure
UP	Unanticipated Problem
US	United States

10.4 PROTOCOL AMENDMENT HISTORY

Version	Date Approved	Description of Change	Brief Rationale
1	3/26/2015	Addition of pilot study phase	To provide feedback on program content
2	4/24/2015	Survey revision and renamed intervention program	Removed certain questions and fixed skip pattern errors in survey. Changed name of program from "Boys Care" to "Manhood 2.0"
3	5/2/2015	Update inclusion criteria	Expanded age range from 14-17 to 14-19 because community partners often work with older youth
4	5/21/2015	Update study docs and update research personnel	Updated parent letter and youth assent to reflect a more flexible program schedule that better fit with community partners' availability. Team member left and was removed
5	6/6/2015	Uploading new study document	Added youth end of program satisfaction survey, which was already in approved protocol but not uploaded
6	7/14/2015	Update study docs	Updates survey measures, recruitment flyers. Feedback forms, study pledge, and changed incentive from gift card to cash because participants may not be able to use gift cares
7	7/21/2015	Update study docs	Parent letter/youth assent minor corrections to align with approved study procedures; baseline survey updated to be more sensitive to LGBTQ participants; EOP survey split into 2 versions depending on exp group
8	10/1/2015	Update inclusion criteria and incentive delivery	Age range changed from 14-19 to 13-19; Each site now has flexibility in incentive delivery (cash, gift card)
9	12/3/2015	Option to have follow-up surveys completed by email/phone	Allowing participants who cannot return to intervention site to complete surveys
10	1/26/2016	Update personnel	New team members added to study team

11	3/3/2016	Update personnel	New team member added to study team
12	6/21/2016	Increased N; added protocol for reached incarcerated participants; modified docs; added new recruitment strategy; update personnel	Increased N to 1000; some participants may become incarcerated during the study and would still like to participate; added recruitment method that paid current participants to bring in new participants; updated parent letters, survey constructs; curriculum; added new team member
14	7/8/2016	Updating survey question	Adding another program choice to the recruit_why1 variable --> specifically whether they are part of the Learn and Earn Program.
15	12/15/2016	Update interview protocol	Add control study participants to the interview protocol; and update interview procedures. Researchers would like to compare interviews between intervention and control participants and learn more about their experiences including bereavement, masculinity and sexual health. New procedures to the interview guide include demographic and screening questionnaire. The questionnaire will be completed by the participant and will customize the questions interviewers ask during the interview. This helps reduce burden of time and effort on the participant by only asking questions relevant to the participant's background. Previously approved assent and interview guide have been updated to align with these new protocols and procedures.
16	1/17/2017	Update personnel	New team members added to study team
17	1/26/2017	Update interview protocol	Added additional questions to the demographic and

			screening questionnaire and a detailed interview script to better standardize the procedures among different interviewers
18	3/15/2017	Uploading letters of support and parent letters	Evidence of permission to conduct research at their sites
19	3/26/2017	Update personnel	Removed members who have left study team
20	4/25/2017	Update personnel	New team members added to study team
21	5/5/2017	Update personnel	New team members added to study team
22	7/3/2017	Adding follow-up letter to participants	To try and reach participants who have not completed their final surveys
23	9/14/2017	Change in personnel; added follow-up contact method	Added new co-I; added Facebook as contact method because other methods may be incomplete or inaccurate
24	11/8/2017	Extending protocol to include crossover study (exploratory)	Extended the study to a few of the participants who have already completed the current research procedures, and asked them to participate in a crossover study. This means that some of the participants who did Manhood 2.0, were offered the control condition (Job Skills Training), and evaluated to see if there are any added benefits to participating in both programs as opposed to just one. To evaluate this, added two more surveys -- a new baseline survey (T4) and a 3 month follow up after the program is completed (T5).
25	4/12/2018	Updated protocol to align all study procedures with approved protocol; update study personnel	Updated team list to those who have left and joined; Based on an internal audit by the department of pediatrics, we have made edits to the protocol to align all study procedures with the approved protocol. Clarifications include adjusting time intervals for surveys to "approximately" 3 months and

			9 months after program ends; # of community sites involved; types of community sites involved; parent letter is provided in English only (no requests for additional languages from community partners); clarification of the verbal assent process and documentation of the verbal assent by research team; uploading the sign in sheet and contact information sheet.
26	6/13/2018	New letter of support; update study personnel	New team member added to study team; LOS added to show working with site/partner
28	7/18/2018	Update study personnel	Removed team members who have left

11 REFERENCES

1. World Health Organization. Prevalence and health effects of intimate partner violence and non-partner sexual violence [Internet]. Italy: World Health Organization; 2013 [cited 2017 Feb 11]. Available from: http://apps.who.int/iris/bitstream/10665/85239/1/9789241564625_eng.pdf

2. Black MC, Basile KC, Breiding MJ, Smith SG, Walters ML, Merrick MT, et al. The National Intimate Partner and Sexual Violence Survey (NISVS):' ' 2010 Summary Report. Atlanta, GA: National Center for Injury Prevention and Control, Centers for Disease Control and Prevention; 2011.

3. Dick RN, McCauley HL, Jones KA, Tancredi DJ, Goldstein S, Blackburn S, et al. Cyber dating abuse among teens using school-based health centers. Pediatrics. 2014 Dec;134(6):e1560–7.

4. Silverman JG, Raj A, Mucci LA, Hathaway JE. Dating violence against adolescent girls and associated substance use, unhealthy weight control, sexual risk behavior, pregnancy, and suicidality. JAMA. 2001 Aug 1;286(5):572–579.

5. Ackard DM, Neumark-Sztainer D, Hannan P. Dating violence among a nationally representative sample of adolescent girls and boys: associations with behavioral and mental health. J Gend Specif Med. 2003;6(3):39–48.

6. Amar AF, Gennaro S. Dating violence in college women - Associated physical injury, healthcare usage, and mental health symptoms. Nur.s Res. 2005;54(4):235–242.

7. Ramisetty-Mikler S, Goebert D, Nishimura S, Caetano R. Dating violence victimization: associated drinking and sexual risk behaviors of Asian, Native Hawaiian, and Caucasian high school students in Hawaii. J Sch Health. 2006 Oct;76(8):423–429.

8. Howard DE, Wang MQ, Yan F. Psychosocial factors associated with reports of physical dating violence among U.S. adolescent females. Adolescence. 2007;42(166):311–324.

9. Champion H, Wagoner K, Song EY, Brown VK, Wolfson M. Adolescent date fighting victimization and perpetration from a multi-community sample: associations with substance use and other violent victimization and perpetration. Int J Adolesc Med Health. 2008;20(4):419–29.

10. Basile KC, Smith SG. Sexual Violence Victimization of Women: Prevalence, Characteristics, and the Role of Public Health and Prevention. Am J Lifestyle Med. 2011 Sep 1;5(5):407–417.

11. Exner-Cortens D, Eckenrode J, Rothman E. Longitudinal associations between teen dating violence victimization and adverse health outcomes. Pediatrics. 2013 Jan;131(1):71–78.

12. Foshee VA, Reyes HLM, Gottfredson NC, Chang L-Y, Ennett ST. A longitudinal examination of psychological, behavioral, academic, and relationship consequences of dating abuse victimization among a primarily rural sample of adolescents. J Adolesc Health. 2013 Dec;53(6):723–729.

13. Decker MR, Peitzmeier S, Olumide A, Acharya R, Ojengbede O, Covarrubias L, et al. Prevalence and Health Impact of Intimate Partner Violence and Non-partner Sexual Violence Among Female

Adolescents Aged 15-19 Years in Vulnerable Urban Environments: A Multi-Country Study. J Adolesc Health. 2014 Dec;55(6 Suppl):S58–67.

14. Tharp AT, DeGue S, Valle LA, Brookmeyer KA, Massetti GM, Matjasko JL. A systematic qualitative review of risk and protective factors for sexual violence perpetration. Trauma Violence Abuse. 2013 Apr;14(2):133–167.

15. Espelage DL, Bosworth K, Simon TR. Examining the social context of bullying behaviors in early adolescence. Journal of Counseling and Development. 2000;78(3):326–333.

16. Espelage DL, Bosworth K, Simon TR. Short-term stability and prospective correlates of bullying in middle-school students: an examination of potential demographic, psychosocial, and environmental influences. Violence Vict. 2001 Aug;16(4):411–426.

17. Berkowitz A. Fostering Men's Responsibility for Preventing Sexual Assault. Washington, D.C: American Psychological Association; 2002.

18. Verma RK, Pulerwitz J, Mahendra V, Khandekar S, Barker G, Fulpagare P, et al. Challenging and Changing Gender Attitudes among Young Men in Mumbai, India. Reprod Health Matters. 2006 Jan;14(28):135–143.

19. Espelage, Dorothy L, Aragon, Steven R, Birkett, Michelle, et al. Homophobic teasing, psychological outcomes, and sexual orientation among high school students: What influence do parents and schools have? School Psychology Review. 2008;37(2):202–216.

20. Reed E, Silverman JG, Raj A, Rothman EF, Decker MR, Gottlieb B, et al. Social and environmental contexts of adolescent and young adult perpetrators of intimate partner violence. Am J Men's Health. 2008;2:260–72.

21. McMahon S, Postmus JL, Koenick RA. Conceptualizing the engaging bystander approach to sexual violence prevention on college campuses. J Coll Stud Dev. 2011;52(1):115–130.

22. Reed E, Silverman JG, Raj A, Decker MR, Miller E. Male perpetration of teen dating violence: associations with neighborhood violence involvement, gender attitudes, and perceived peer and neighborhood norms. J Urban Health. 2011 Apr;88(2):226–239.

23. Banyard VL, Plante EG, Moynihan MM. Bystander education: Bringing a broader community perspective to sexual violence prevention. J Community Psychol. 2004 Jan;32(1):61–79.

24. Foubert JD, Perry BC. Creating lasting attitude and behavior change in fraternity members and male student athletes: the qualitative impact of an empathy-based rape prevention program. Violence Against Women. 2007 Jan;13(1):70–86.

25. Tulloch T, Kaufman M. Adolescent sexuality. Pediatr Rev. 2013 Jan;34(1):29–37; quiz 38.

26. Marques M, Ressa N. The Sexuality Education Initiative: a programme involving teenagers, schools, parents and sexual health services in Los Angeles, CA, USA. Reprod Health Matters. 2013 Jan;21(41):124–135.

27. Kimmel A, Williams TT, Veinot TC, Campbell B, Campbell TR, Valacak M, et al. I make sure I am safe and I make sure I have myself in every way possible ': African-American youth perspectives on sexuality education. Sex Educ. 2013 Mar 1;13(2):172–185.

28. Haberland NA. The case for addressing gender and power in sexuality and HIV education: a comprehensive review of evaluation studies. Int Perspect Sex Reprod Health. 2015 Mar;41(1):31–42.

29. Barker G, Ricardo C, Nascimento M. Engaging men and boys in changing gender-based inequity in health: evidence from programme interventions. Geneva, Switzerland: World Health Organization; 2007. Report No.: 9 789241 595490.

30. Jewkes R, Nduna M, Levin J, Jama N, Dunkle K, Puren A, et al. Impact of Stepping Stones on incidence of HIV and HSV-2 and sexual behaviour in rural South Africa: cluster randomised controlled trial. Br Med J. 2008;337(7666).

31. Pulerwitz J, Martin S, Mehta M, Castillo T, Kidanu A, Verani F, et al. Promoting gender equity for HIV and violence prevention: results from the PEPFAR Male Norms Initiative Evaluation in Ethiopia. Washington, DC: PATH. 2010;

32. Kim JC, Watts CH, Hargreaves JR, Ndhlovu LX, Phetla G, Morison LA, et al. Understanding the impact of a microfinance-based intervention on women's empowerment and the reduction of intimate partner violence in South Africa. Am J Public Health. 2007 Oct;97(10):1794–1802.

33. Tancredi DJ, Silverman JG, Decker MR, McCauley HL, Anderson HA, Jones KA, et al. Cluster randomized controlled trial protocol: Addressing reproductive coercion in health settings (ARCHES). BMC Womens Health. 2015 Aug 6;15:57.

34. Miller E, Tancredi DJ, McCauley HL, Decker MR, Virata MCD, Anderson HA, et al. Coaching Boys into Men": A cluster-randomized controlled trial of a dating violence prevention program. J Adolesc Health. 2012 Nov;51(5):431–438.

35. Dartnall E, Jewkes R. Sexual violence against women: The scope of the problem. Best Pract Res Clin Obstet Gynaecol. 2013 Feb;27(1):3–13.

36. Koss MP, Gidycz CA. Sexual experiences survey: Reliability and validity. J Consult Clin Psychol. 1985 Jun;53(3):422–423.

37. AAUW Educational Foundation Sexual Harassment Task Force. Harassment-free hallways: how to stop sexual harassment in schools [Internet]. 2001. Available from: http://www.aauw.org/learn/research/upload/completeguide.pdf

38. Espelage DL, Holt MK. Dating violence & sexual harassment across the bully-victim continuum among middle and high school students. J Youth Adolesc. 2007 Jun 16;36(6):799–811.

39. Ybarra ML, Espelage DL, Mitchell KJ. The co-occurrence of Internet harassment and unwanted sexual solicitation victimization and perpetration: Associations with psychosocial indicators. J Adolesc Health. 2007 Dec;41(6 Suppl 1):S31–41.

40. Bennett DC, Guran EL, Ramos MC, Margolin G. College students' electronic victimization in friendships and dating relationships: anticipated distress and associations with risky behaviors. Violence Vict. 2011;26(4):410–429.

41. Brafford LJ, Beck KH. Development and validation of a condom self-efficacy scale for college students. J Am Coll Health. 1991 Mar;39(5):219–225.

42. Cabral RJ, Galavotti C, Stark MJ, Gargiullo PM, Semaan S, Adams J, et al. Psychosocial factors associated with stage of change for contraceptive use among women at increased risk for HIV and stds. J Appl Soc Psychol. 2004 May;34(5):959–983.

43. Aalsma MC, Carpentier MY, Azzouz F, Fortenberry JD. Longitudinal effects of health-harming and health-protective behaviors within adolescent romantic dyads. Soc Sci Med. 2012 May;74(9):1444–1451.

44. Borrero S, Farkas A, Dehlendorf C, Rocca CH. Racial and ethnic differences in men's knowledge and attitudes about contraception. Contraception. 2013 Oct;88(4):532–538.

45. Carvajal DN, Ghazarian SR, Shea Crowne S, Bohrer Brown P, Carter Pokras O, Duggan AK, et al. Is depression associated with contraceptive motivations, intentions, and use among a sample of low-income Latinas? Womens Health Issues. 2014 Feb;24(1):e105–13.

46. Rothman EF, Decker MR, Silverman JG. Evaluation of a teen dating violence social marketing campaign: Lessons learned when the null hypothesis was accepted. New Directions for Evaluation. 2006;2006(110):33–44.

47. Pulerwitz J, Barker G. Measuring attitudes toward gender norms among young men in Brazil: Development and psychometric evaluation of the GEM scale. Men Masc. 2007 May 18;10(3):322–338.

48. Chu JY, Porche MV, Tolman DL. The adolescent masculinity ideology in relationships scale. Men Masc. 2005 Jul;8(1):93–115.

49. Lorimer K, Gray CM, Hunt K, Wyke S, Anderson A, Benzeval M. Response to written feedback of clinical data within a longitudinal study: a qualitative study exploring the ethical implications. BMC Med Res Methodol. 2011 Jan 27;11:10.

50. Miller E, Tancredi DJ, McCauley HL, Decker MR, Virata MCD, Anderson HA, et al. One-year follow-up of a coach-delivered dating violence prevention program: A cluster randomized controlled trial. Am J Prev Med. 2013 Jul;45(1):108–112.

51. Twisk J, Proper K. Is analysis of covariance the most appropriate way to analyse changes in randomized controlled trials? J Clin Epidemiol. 2005 Feb;58(2):211–212.

52. Miller E, Goldstein S, McCauley HL, Jones KA, Dick RN, Jetton J, et al. School Health Center Healthy Adolescent Relationships Program (SHARP): A Cluster Randomized Controlled Trial. 2014;

53. Kraemer HC, Kiernan M, Essex M, Kupfer DJ. How and why criteria defining moderators and mediators differ between the Baron & Kenny and MacArthur approaches. Health Psychol. 2008 Mar;27(2S):S101–8.
