# Supplementary material for: Effect of a Community-Based Gender Norms Program on Sexual Violence Perpetration by Adolescent Boys and Young Men: A Cluster Randomized Clinical Trial
Source: JAMA Netw Open. 2020 Dec 22;3(12):e2028499. doi: 10.1001/jamanetworkopen.2020.28499 (PMC7756236; doi:10.1001/jamanetworkopen.2020.28499)
Supplement: Supplement 2. — eFigure. Manhood 2.0: Engendering Healthy Masculinities Study Flow eTable. Demographic and Secondary Outcome Characteristics Between Study Participants With Complete and Missing Data for the Primary Outcome (Attrition Analysis) [file jamanetwopen-e2028499-s002.pdf]

Supplemental Online Content

Miller E, Jones KA, Culyba AJ, et al. Effect of a community-based gender norms program on sexual violence perpetration by adolescent boys and young men: a cluster randomized clinical trial. *JAMA Netw Open*. 2020;3(12):e2028499. doi:10.1001/jamanetworkopen.2020.28499

eFigure. Manhood 2.0: Engendering Healthy Masculinities Study Flow
eTable. Demographic and Secondary Outcome Characteristics Between Study Participants With Complete and Missing Data for the Primary Outcome (Attrition Analysis)

This supplemental material has been provided by the authors to give readers additional information about their work.

© 2020 Miller E et al. *JAMA Network Open.*

eTable 1. Demographic and Secondary Outcome Characteristics Between Study Participants With Complete and Missing Data for the Primary Outcome (Attrition Analysis)

	**Primary Outcome Present?**		**p-value[b]**
	**Yes (n=622) No. (%)[a]**	**No (n=244) No. (%)[a]**	
**Demographics**			
Age in years, mean (SD)	15.4 (1.6)	15.7 (1.8)	0.070
Race			
Black/African American	447 (75%)	163 (73%)	0.25
White	19 (3%)	10 (5%)	
Hispanic	31 (5%)	22 (10%)	
Multiracial	42 (7%)	13 (6%)	
Other	54 (9%)	16 (7%)	
Born in the United States			
Yes	35 (6%)	14 (6%)	0.93
No	549 (94%)	210 (94%)	
Education status			
Currently in school	528 (91%)	207 (92%)	0.71
Not in school – completed high school degree	19 (3%)	9 (4%)	
Not in school – did not complete high school degree	33 (6%)	9 (4%)	
Current grade level[c]			
8th	114 (22%)	49 (25%)	0.15
9th	131 (26%)	49 (25%)	
10th	117 (23%)	34 (17%)	
11th	93 (18%)	37 (19%)	
12th	49 (10%)	23 (12%)	
Finished HS or Received GED	4 (1%)	5 (3%)	
College	3 (1%)	3 (2%)	
Juvenile justice-involved			
Yes	54 (9%)	49 (20%)	**0.0003**
No	569 (91%)	194 (80%)	
Parents' / guardians' highest education			
Did not complete high school	273 (47%)	105 (48%)	0.1203
Completed high school or GED	101 (17%)	48 (22%)	
Some college	52 (9%)	14 (6%)	
College degree or higher	156 (27%)	53 (24%)	
**Secondary Outcomes**			
Gender Equitable Attitudes, mean (SD)	3.4 (0.51)	3.31 (0.49)	**0.030**
Recognition of Abuse, mean (SD)	3.1 (1.1)	3.0 (1.1)	0.15
Intention to Intervene, mean (SD)	2.5 (1.2)	2.6 (1.2)	0.33
Condom Negotiation Self-Efficacy, mean (SD)	3.5 (0.7)	3.5 (0.68)	0.79
SV/ARA Summary Score, mean (SD)	2.7 (3.6)	2.7 (3.6)	0.99
Positive Bystander Behavior	0.6 (1.2)	0.7 (1.2)	0.97
Negative Bystander Behavior	1.4 (2.0)	1.5 (2.0)	0.68

[a]Percentages may not sum to 100 due to small amounts of missing data
[b]Wald log-linear chi-squared (categorical variables), linear regression (continuous variables) were used to calculate p values, all accounting for neighborhood-level clustering

© 2020 Miller E et al. *JAMA Network Open.*

[c]Among those currently in school

© 2020 Miller E et al. *JAMA Network Open.*

eFigure. Manhood 2.0: Engendering Healthy Masculinities Study Flow

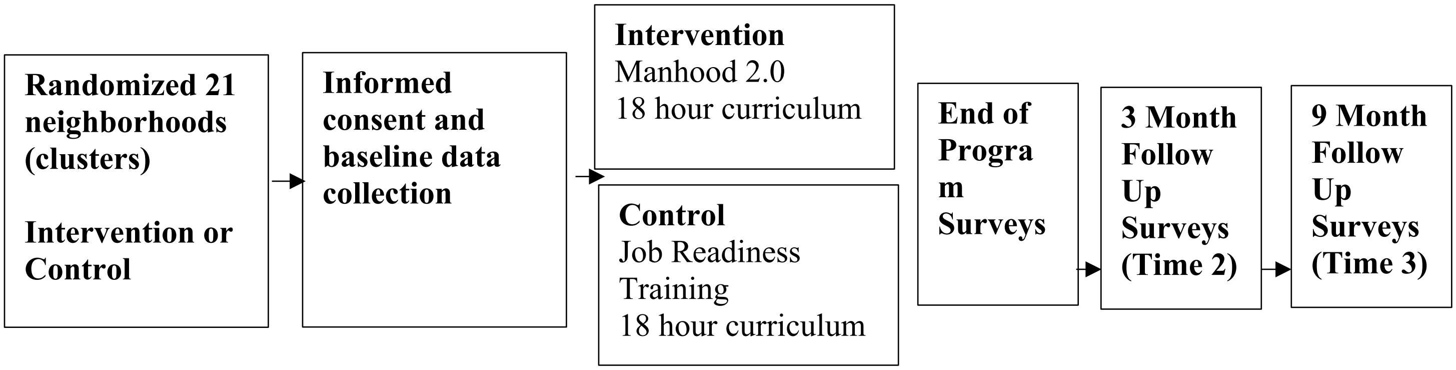

© 2020 Miller E et al. *JAMA Network Open.*
