# Supplementary material for: Effect of a Community-Based Gender Norms Program on Sexual Violence Perpetration by Adolescent Boys and Young Men: A Cluster Randomized Clinical Trial
Source: JAMA Netw Open. 2020 Dec 22;3(12):e2028499. doi: 10.1001/jamanetworkopen.2020.28499 (PMC7756236; doi:10.1001/jamanetworkopen.2020.28499)
Supplement: Supplement 3. — Data Sharing Statement [file jamanetwopen-e2028499-s003.pdf]

Data Sharing Statement

Miller. Effect of a community-based gender norms program on sexual violence perpetration by adolescent boys and young Men. *JAMA Netw Open*. Published December 22, 2020. doi:10.1001/jamanetworkopen.2020.28499

Data

Data available: Yes
Data types: Deidentified participant data, Data dictionary
How to access data: Please contact Elizabeth Miller to request these data, elizabeth.miller@chp.edu.
When available: beginning date: 06-01-2021

Supporting Documents

Document types: None

Additional Information

Who can access the data: Researchers whose proposed use of the data has been approved.
Types of analyses: For a specified purpose, to be approved by the collaborators.
Mechanisms of data availability: After approval of a proposal and with a signed data access agreement.
